# FAK Acts as a Suppressor of RTK-MAP Kinase Signalling in *Drosophila melanogaster* Epithelia and Human Cancer Cells

**DOI:** 10.1371/journal.pgen.1004262

**Published:** 2014-03-27

**Authors:** Juan Pablo Macagno, Jesica Diaz Vera, Yachuan Yu, Iain MacPherson, Emma Sandilands, Ruth Palmer, Jim C. Norman, Margaret Frame, Marcos Vidal

**Affiliations:** 1 Cancer Research UK Beatson Institute, Garscube Estate, Glasgow, United Kingdom; 2 Edinburgh Cancer Research UK Centre, Institute of Genetics and Molecular Medicine, University of Edinburgh, Western General Hospital, Edinburgh, United Kingdom; 3 Department of Molecular Biology, Umeå University, Umeå, Sweden; University of Washington, United States of America

## Abstract

Receptor Tyrosine Kinases (RTKs) and Focal Adhesion Kinase (FAK) regulate multiple signalling pathways, including mitogen-activated protein (MAP) kinase pathway. FAK interacts with several RTKs but little is known about how FAK regulates their downstream signalling. Here we investigated how FAK regulates signalling resulting from the overexpression of the RTKs RET and EGFR. FAK suppressed RTKs signalling in *Drosophila melanogaster* epithelia by impairing MAPK pathway. This regulation was also observed in MDA-MB-231 human breast cancer cells, suggesting it is a conserved phenomenon in humans. Mechanistically, FAK reduced receptor recycling into the plasma membrane, which resulted in lower MAPK activation. Conversely, increasing the membrane pool of the receptor increased MAPK pathway signalling. FAK is widely considered as a therapeutic target in cancer biology; however, it also has tumour suppressor properties in some contexts. Therefore, the FAK-mediated negative regulation of RTK/MAPK signalling described here may have potential implications in the designing of therapy strategies for RTK-driven tumours.

## Author Summary

Due to their deregulation in cancer and their potential to be inhibited by small chemical compounds, tyrosine kinases are among the most important targets under consideration for cancer therapeutics. One such oncogenic tyrosine kinase is FAK, which is known to regulate cellular signalling downstream of Integrins and Receptor Tyrosine Kinases (RTK) at the cell surface. In this study, however, we report that FAK can act as a suppressor of oncogenic Receptor Tyrosine Kinases. This mechanism was observed in fruit fly tissues *in vivo* and human cancer-derived cells *in vitro*, which additionally suggests it is an evolutionary conserved mechanism in humans. FAK mediated this inhibition by controlling the sub-cellular localisation of receptors, via suppression of receptor recycling to the cell surface. These results suggest that in some particular cancer contexts such as RTK-driven tumours, FAK may act as a tumour suppressor and therefore, may not be a valid drug target.

## Introduction

Research in model organisms can provide important insights on the effects of oncogenic pathways in different *in vivo* environments [1], [2]. Particularly, *Drosophila melanogaster* has made numerous contributions to cancer biology, *e.g.* by identifying components of several signalling pathways such as the Hippo [3] and RTK/Ras/MAPK signalling pathways [4]–[7].

FAK is a cytoplasmic non-receptor tyrosine kinase that interacts primarily with Integrins at the focal adhesion sub-domains of the plasma membrane (reviewed in [8]). FAK belongs to a hub where phosphorylation signals are regulated and transferred into the cell, therefore it is implicated in many cellular processes such as adhesion, migration, survival and differentiation [8], [9], and is normally found over-expressed in migrating and invasive tumour cells [10]. The current knowledge suggests that abnormal FAK activation is a key driver of tumour cell motility and survival in conditions that would trigger anoikis (detachment-dependent apoptosis) (reviewed in [10], [11]). Thus, FAK has been regarded as a potential target for cancer therapeutics.

In *Drosophila*, there is a single FAK homologue (*FAK56D*, here aftercalled *dFAK* [12]–[14]); dFAK is ubiquitously expressed, with particularly high levels in the developing Central Nervous System (CNS) and muscle [14]. Consistently, *dFAK* mutants have abnormal neuromuscular junction growth and optic stalk structure [15], [16]. Nevertheless, *dFAK* mutants are viable and fertile [17], proving it is dispensable for general development. This suggests the role of dFAK may become apparent only under conditions of stress. In fact, *dFAK* mutants display sensitivity to mechanical stimuli, suffering seizure and temporal paralysis [18].

As oncogene activation is a condition of stress, we examined the role of dFAK within a context of a *Drosophila* model of cancer [19]–[21] achieved by the expression of the receptor tyrosine kinase RET (Rearranged during transformation). Activating mutations in *RET* cause the familial cancer syndrome Multiple Endocrine Neoplasia type 2 (MEN2) (reviewed in [22], [23]). Furthermore, chromosomal translocations implicating ectopic expression of RET are frequent in Papillary thyroid Carcinoma (PTC), the most common type of thyroid cancer [24], [25], pheochromocytomas [26] and breast carcinoma [27].

Several RTKs were described to directly phosphorylate and activate FAK [28], [29]. Interestingly, direct interaction and mutual phosphorylation between FAK and RET has also been reported [30], [31]. Nevertheless, the functional importance of FAK in RTK signalling *in vivo*, particularly in the context of tumour development, is not yet clear [29] (reviewed in [11]). Therefore, dFAK is a likely candidate to be activated by *Drosophila* RET (here after called dRET) and mediate its signalling cascade in *Drosophila*.

In this study, we investigated the regulatory role of FAK downstream of RTKs in epithelial tissues. Unexpectedly, our findings demonstrate that FAK constitutes a negative regulator of RET and also, the RTK epithelial growth factor receptor (EGFR). FAK impairs RTK signalling specifically via the Ras/MAPK pathway, which in turn impacts on the survival and differentiation outcomes of RTK-expressing tissues. Because FAK is currently considered an oncogenic target, our study may have implications in future cancer therapies. Our results suggest that targeting FAK in the context of some RTK-driven tumours might be detrimental rather than beneficial for the host.

## Results

### RET activates FAK and MAPK

**Figure 1:**
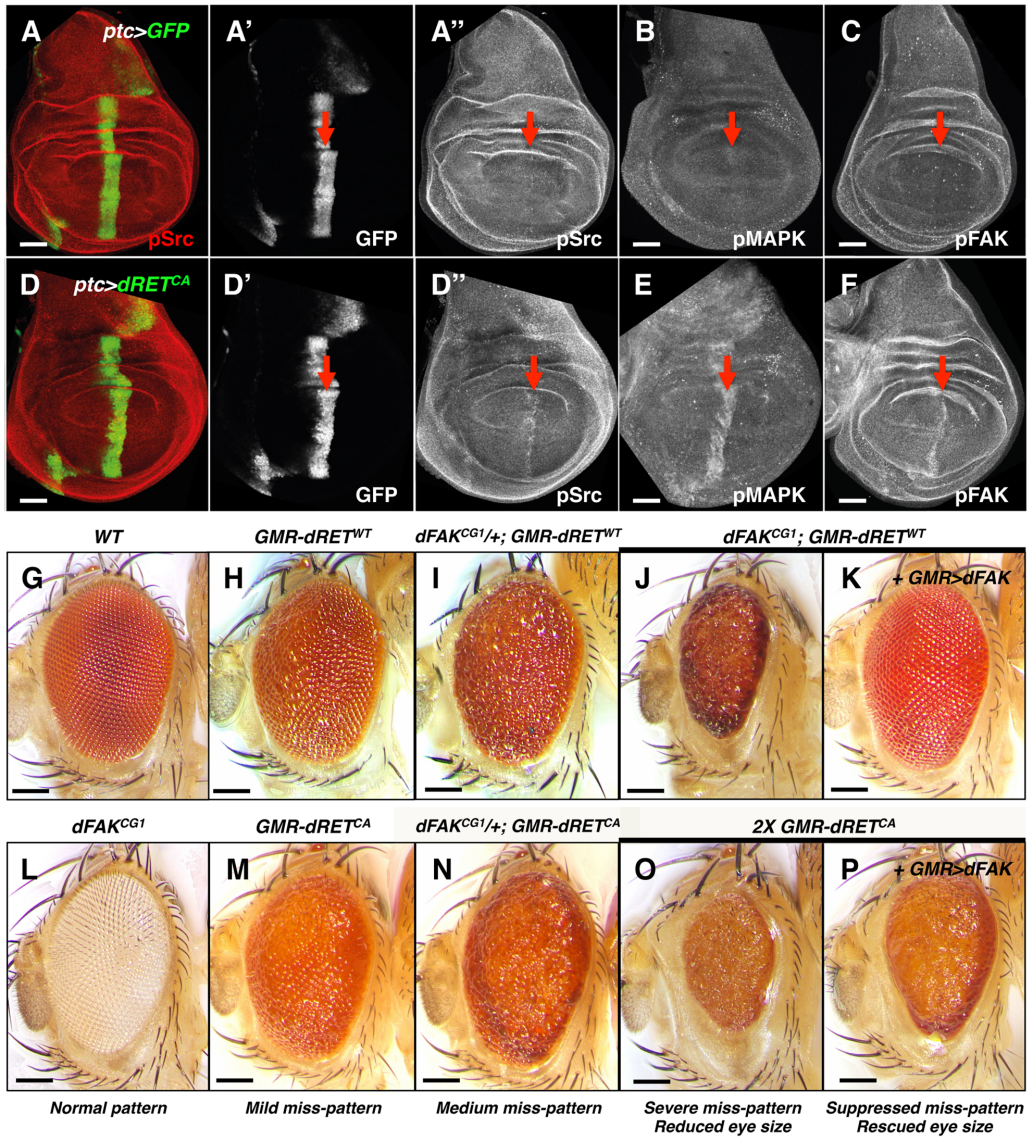
Genetic interactions between RET and FAK. (A–F) Confocal images of wing disc epithelia. Control tissues (A–C) and experimental tissues (D–F) expressed GFP driven by *ptc-gal4*, shown in A′ and D′. Experimental tissues (D–F) also expressed dRET^CA^. Immunostaining against pSrc (A″ and D″), pMAPK (B and E) and pFAK proteins (C and F; see methods), as a proxy for probing their activation levels, are shown in grayscale panels. Note increased phosphorylation of Src, MAPK and FAK after dRET^CA^ expression within the *ptc* domain, indicated by red arrows. Scale bars, 50 µm. (G–P) Images of adult eyes with indicated relevant genotypes; full genotypes are listed in supplemental material. GMR (glass multimer reporter) is an eye specific promoter. *GMR-gal4* was used to drive *UAS-dFAK* transgene expression. *GMR-dRET^WT^* and *GMR-dRET^CA^* are fusion recombinant constructs. *Wild type* (G) and *dFAK^CG1^* (L) animals displayed a normal eye pattern; note that the *dFAK^CG1^* is in a *white* background (see Figure S1B). Expression of *dRET^WT^* caused a mild eye miss-patterning phenotype (H), and lowering the genetic dose of *dFAK* gene in these animals either enhanced eye roughness (I–J) or completely disrupted patterning and decreased eye size (J). Reciprocally, suppression of both effects was observed by co-expression of dFAK (K). (M–N) A similar enhancement was observed by halving the dose of *dFAK* gene after expression of dRET^CA^. (O) Doubling dRET^CA^ dose caused a very rough and small eye, comparable to (J), which was partially suppressed when dFAK was also expressed (P). Eye size quantifications of panels G, H, J, K, O and P are shown in Figure S1D. Scale bars, 100 µm.

We expressed a constitutively activated form of dRET (hereafter called dRET^CA^) with the *ptc-gal4* driver, which is active in a stripe of cells immediately anterior to the Anterior/Posterior compartmental boundary of the developing wing imaginal discs (Figure 1A and 1D). GFP expression by itself did not affect this cell population nor produced activation of the cytoplasmic kinases Src, FAK or MAPK (Figure 1A–C). As expected from previous studies [19], expression of dRET^CA^ (*ptc>dRET^CA^*) led to phosphorylation of Src and MAPK on residues that report their activation (Figure 1D–E, see methods). Interestingly, we also observed increased phosphorylation of FAK (Figure 1F).

Next, we tested genetically the importance of Src, Ras/MAPK and FAK downstream of RET. The *Drosophila* compound eye is an elegant structure composed of about 750 hexagonal units called ommatidia, which pattern in a honeycomb-shaped array (Figure 1G) [32]. This repetitive array makes the eye very sensitive to perturbations in signalling pathways. The ectopic expression of a single wild type copy of the *Drosophila RET* gene (hereafter called *dRET^WT^*) under the direct control of the eye-specific GMR promoter (*GMR-dRET^WT^*) disturbed the normal array of ommatidia, creating a ‘rough’ eye phenotype (Figure 1H; [19]). In a search of genes involved in dRET signalling, previously known members of the Ras/MAPK and Src signalling pathways were identified (*drk* (Grb2), *Sos*, *Ras85D*, *ksr*, *Gap1* (RasGAP), *Src42A*, *Src64B*, *Jra* (c-Jun) and *basket* (JNK) [19]. As expected, we observed that the dRET-induced phenotype was suppressed when Src42A or Ras85D proteins were knocked down by RNA interference (Figure S2A), confirming that they play key roles downstream of dRET signalling.

Eyes from *dFAK^CG1^* null mutants displayed normal patterning (Figure 1L). Interestingly, and contrary to expectations of a potential dRET-signalling effector, loss of FAK did not suppress the rough eye phenotype. In fact, hetero- and homozygosis of *dFAK^CG1^* either enhanced the miss-patterning or caused a smaller eye due to a completely disrupted ommatidial pattern, respectively (Figure 1I and 1J).

To prove that only the loss of *dFAK* was responsible for the phenotype observed in *dFAK^CG1^; GMR-dRET^WT^* animals (Figure 1J) and no other genetic background mutation was influencing the results, we next used two additional *dFAK* mutant strains (Figure S1A). These strains bear a P-element insertion within *dFAK* and by measuring the expression levels of *dFAK* mRNA transcripts were confirmed to be hypomorphic lines (Figure S1B). Different *dFAK* allelic combinations showed a similar genetic interaction with *GMR-dRET^WT^* (Figures S1C).

The latter phenotype was comparable to the effect mediated by high expression of dRET^CA^ in the eye (Figure 1O). dFAK overexpression rescued the effects of *dFAK* null mutation (Figure 1K) and partially restored the small size induced by the constitutively active RET isoform (compare Figure 1O with 1P; eye sizes are quantified in S1D).

Overall, these data suggest that dRET activates dFAK, which in turn may have unanticipated suppressive effects on RET signalling.

### FAK suppresses RET in different fly epithelia

**Figure 2:**
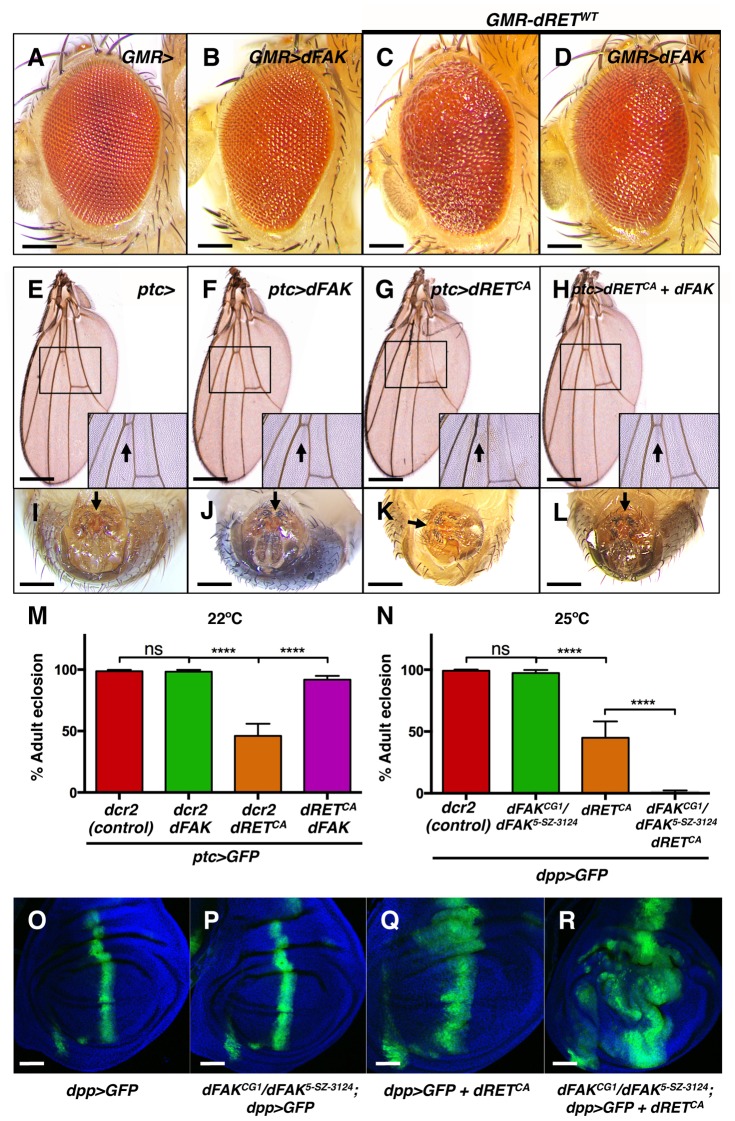
FAK suppresses RET-driven effects in different fly tissues. (A–D) Eyes expressing dFAK displayed a normal adult eye phenotype, while dRET^WT^ expression perturbed the normal pattern. Co-expression of dRET^WT^ and dFAK supressed dRET-driven mis-patterning defects. Scale bar, 100 µm. (E–H) dFAK-expressing wings via *ptc-gal4* showed no detectable defects similarly to control wings. Expression of dRET^CA^ led to disappearance of anterior cross veins in all adult escapers (arrow in inset box), which was rescued by simultaneous expression of dFAK with full genetic penetrance. Scale bar, 500 µm. (I–L) *ptc*-driven dRET^CA^ expression also led to incomplete rotation of the male genitalia in all adult escapers (arrows). dFAK co-expression rescued this phenotype with full penetrance and it did not affect the normal development of the genitalia by itself. Scale bar, 100 µm. (M) Quantification of the penetrance of adult eclosion for the indicated genotypes, note that dFAK co-expression rescued significantly the developmental lethality of *ptc>dRET^CA^* animals. Error bars are standard deviation in this and all plots; ‘ns’ stands for non-statistically significant, **** means p<0.0001 in this and all plots (see methods). (N) Conversely, *dFAK* loss, which by itself had no effect in viability, enhanced to almost full penetrance the developmental lethality of *dpp>dRET^CA^* animals. (O–R) Confocal images from wing discs with the indicated genotypes. Note that *dFAK* mutation enhanced the size and shape defects associated with ectopic expression of *dRET^CA^* within the *dpp* stripe. For a detailed characterisation of the *dFAK* mutant alleles used here, please see Figure S1A–B. Scale bars, 50 µm.

To further test whether FAK can inhibit RET signalling we combined dRET and dFAK in different imaginal discs using the *GMR-gal4, dpp-gal4* and *ptc-gal4* drivers. dFAK overexpression by itself had no apparent phenotype on adult survival and tissue patterning (Figure 2A, B, E, F, I, J, M).

However, dRET expression did affect several tissues of the adult fly. As mentioned above, dRET^WT^ expression in the eye altered the normal pattern of ommatidia (Figure 2C, see also 1H), which was prevented when dFAK was simultaneously expressed (Figure 2D). All escaper *ptc>dRET^CA^* adults showed wing vein defects [33], with absence of the anterior cross vein, which lies within the *ptc* domain of the wing anlage (Figure 2G). Remarkably, this phenotype was rescued by the co-expression of dFAK and dRET^CA^ (Figure 2H). All escaper *ptc>dRET^CA^* adult males also displayed rotation defects in the epandrium (Figure 2K). The *patched* gene is expressed in a compartment-specific manner across most imaginal discs in the larva, including the genital disc [34]. Therefore, dRET expression in this tissue perturbed normal development and rotation of the male genital organ of adult escapers [35], [36]. Importantly, dFAK also suppressed this dRET-induced phenotype and restored the proper orientation of the male genitalia (Figure 2L).

Additionally, dRET expression also resulted in developmental toxicity (with a penetrance that depended on the temperature and other factors [33]). Correspondingly, 46% of *ptc>dRET^CA^*-expressing flies made it to adulthood (n = 187) in our experimental conditions. Co-expression of dRET^CA^ and dFAK increased survival up to 92% (n = 106) (Figure 2M). Conversely, loss of dFAK enhanced the morphological defects induced by dRET in the imaginal discs (Figure 2O–R) and reduced the survival rate of *dpp>dRET^CA^* animals (Figure 2N).

Together, these observations demonstrate that dFAK inhibits dRET-induced phenotypic effects in multiple imaginal disc epithelia.

### The N-terminal domain of FAK, but not its kinase activity, is required to suppress RET

**Figure 3:**
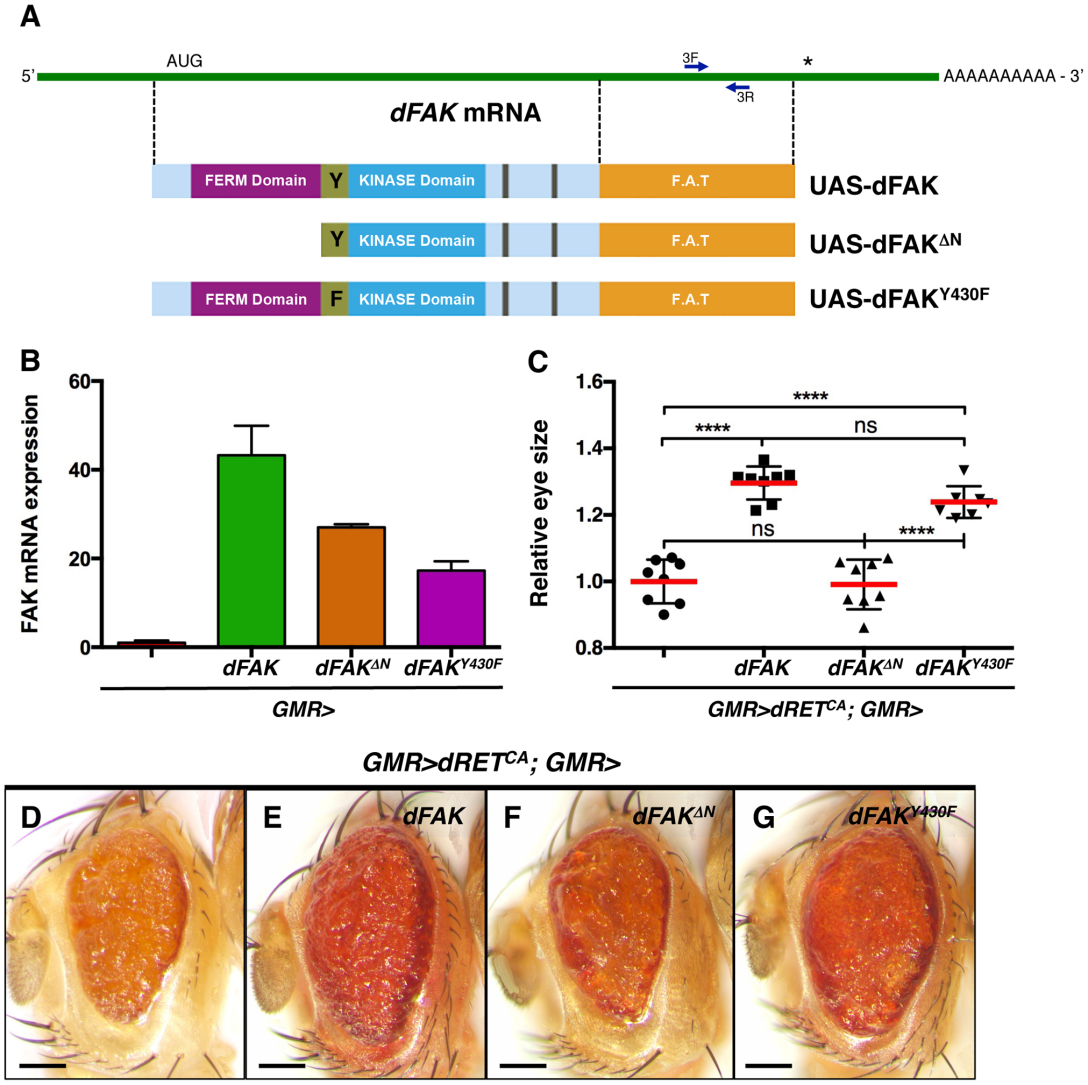
Requirement of the N-terminal FAK FERM domain. (A) Linear representation of *dFAK* mRNA, its derivatives UAS-transgenes and their resultant protein isoforms: a full-length dFAK isoform; an N-terminal deletion mutant that lacks the first 400 amino acids residues of dFAK including its FERM domain; and a point mutant isoform that bears a replacement of the Tyrosine^430^ residue for a Phenylalanine residue, which impairs the auto-phosphorylation site and consequently the kinase activity of dFAK. (B) Expression profiles of each *UAS-dFAK* transgene in the eye (driven by *GMR-gal4*) as determined by quantitative (q) PCR of RNA samples (see methods). We used a pair of primers (3F and 3L) flanking a 200 bp region corresponding to the C-terminal domain (FAT: Focal adhesion targeting domain), which is a common region to all the isoforms. (C) Eye size quantification of the indicated genotypes, shown in D-G. Eye sizes on the Y-axis are represented as relative values to the mean of *GMR>dRET^CA^* (‘ns’: not statistically significant; **** = p<0.0001; n = 8–10 for each genotype). (D–G) Eye micrographs correspond to the indicated genotypes. Note that while the auto-phosphorylation mutant version of dFAK was expressed at lower levels than the N-terminal mutant isoform (B), it was still able to rescue the size of dRET^CA^-expressing eyes (G), to a similar extent as the full-length dFAK isoform (E). However, the N-terminal deletion mutant isoform did not suppress the small eye size of dRET^CA^ animals (C, D and F). Scale bar, 100 µm.

To gain insight into the mechanism by which FAK suppresses RET signalling, we co-expressed dRET^CA^ and different dFAK mutant isoforms in the eye (schematised in Figure 3A). When driven by *GMR-gal4*, the transcript levels from these *UAS-dFAK* transgenes ranged from 20- to 40-fold over the endogenous levels of *dFAK* transcript (Figure 3B). When expressed simultaneously with dRET^CA^ and by measuring the eye areas of the consequent phenotypes (Figure 3C) we observed that a wild type allele of dFAK significantly restored the eye size when compared to control *GMR>dRET^CA^* eyes (Figure 3D and 3E). Interestingly, and in correlation with observations made in different systems [30], [31], the N-terminal FERM domain of dFAK appears important for this functional interaction between dFAK and dRET: expression of a N-terminal deletion mutant (dFAK^ΔN^) failed to modify the eye size of *GMR>dRET^CA^* flies (Figure 3C and 3F). On the other hand, a point mutant isoform in dFAK major auto-phosphorylation site (FAK^Y430F^, equivalent to tyrosine Y-397 in human FAK), did significantly rescue the eye size of *GMR>dRET^CA^* flies and the patterning of *FAK^CG1^/+; GMR>dRET^WT^* eyes (Figure 3G and S2C). These results suggest that the FERM domain of dFAK is necessary to inhibit RET signalling, while the major autophosphorylation site— required for full FAK kinase activity [37]— may not be essential. Some systems require FAK as a scaffold protein but not as a kinase [38] and our results suggest that this is the case in the functional interaction with RET.

### Moderate RET/FAK ratios suppress apoptosis

**Figure 4:**
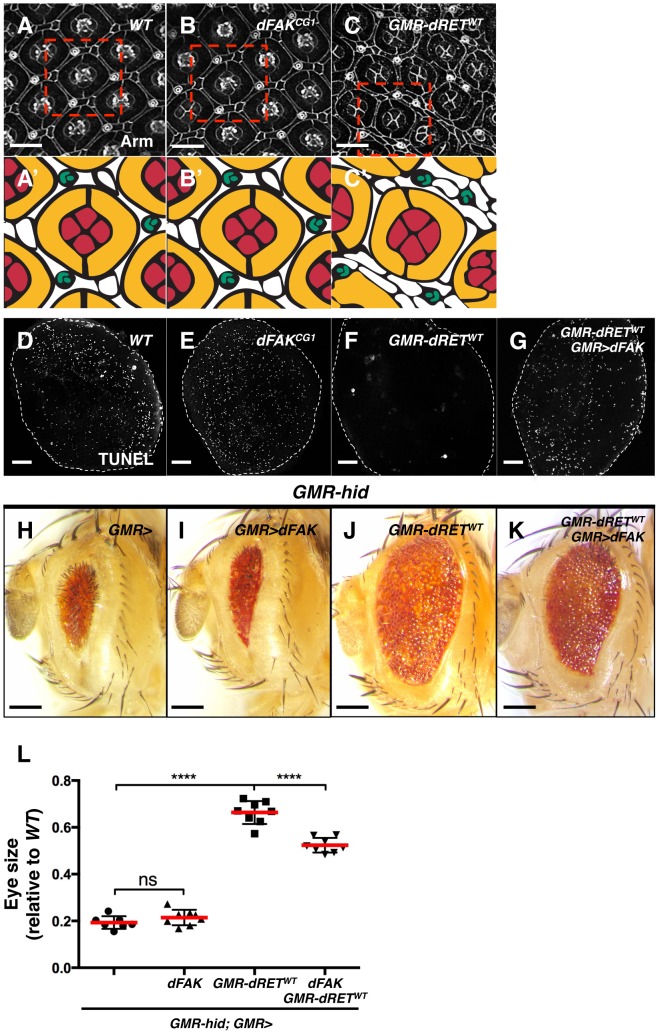
Moderate relative RET/FAK levels lead to inhibition of programmed cell death. (A–C) Armadillo immunostaining revealed cell outlines of *wild type* (A), *dFAK^CG1^* (B), and *GMR-dRET^WT^* (C) retinas at 42 hs after puparium formation (APF). The boxed areas were traced to highlight their cellular composition (A′–C′). Each ommatidium is composed of 4 cone cells (red), 2 primary pigments cells (yellow), 6 secondary and three tertiary cells (white), and three-bristle cells (green) make the hexagonal lattice. Note that *dFAK^CG1^* eyes display normal patterning (B′) while *GMR-dRET^WT^* retinas displayed normal ommatidial cores but additional interommatidial cells (white cells in C′). Scale bars, 10 µm. (D–G) TUNEL labelling of retinas at 28 h APF. Note that the developmental programmed cell death observed in *wild type* and *dFAK^CG1^* retinas were suppressed in *GMR-dRET^WT^* retinas. Co-expression of dFAK rescued this inhibition of cell death (G). Scale bar, 50 µm. (H–K) Hid overexpression (*GMR-hid*) gave a small eye phenotype, which was suppressed by dRET^WT^ co-expression (J). This dRET-dependent inhibition was also suppressed by dFAK co-expression (K) while dFAK itself did not suppress Hid-mediated effects in the eye (I). Scale bar, 100 µm. (L) Eye size quantification of the indicated genotypes (as depicted in panels H–K) represented as relative values to the *wild type* mean value (‘ns’: not statistically significant; **** = p<0.0001; n = 8–10 for each genotype).

Next, we further characterized the RET/FAK regulatory loop and its signalling output; specifically, we analysed how different experimental conditions that altered relative RET/FAK levels affected eye patterning. To gain further insights on how different RET/FAK ratios influence tissue cell fate *in vivo*, we examined the cell composition of the eye tissue. Each ommatidial unit consists of eight photoreceptor and six supporting cells (four cone cells and two primary pigment cells) (Figure 4A) [32] while a hexagonal lattice of secondary and tertiary pigment cells surrounds the units (white coloured in Figure 4A′–C′). Photoreceptor cell clusters are specified first; they constitute ‘organizing centres’ that instruct neighbouring cells to differentiate into cone cells and primary pigment cells. The hexagonal lattice patterns by local cell reorganization and elimination of surplus cells via a wave of developmental programmed cell death.

We visualized the final pattern of retinas at 42 hs after puparium formation (APF). *dFAK* mutant retinas were indistinguishable from their wild type counterparts (Figure 4A and 4B). When dRET^WT^ was expressed by itself, the ommatidial cores remained normal, with four cone cells surrounded by two primary pigment cells. Nevertheless, eye patterning was altered, displaying supernumerary interommatidial cells (Figure 4C). This suggested that developmental programmed cell death might be suppressed during eye development. Indeed, while wild type or *FAK^CG1^* retinas displayed a large number of apoptotic cells at 28 h APF —a time of high levels of developmental apoptotic cell death— there were virtually no apoptotic cells in *GMR-dRET^WT^* retinas (Figures 4D–F). Remarkably, developmental cell death was rescued by co-expression of dRET^WT^ and dFAK, further demonstrating the ability of dFAK to suppress dRET signalling (Figure 4G).

Programmed cell death during eye development is mainly dependent on the pro-apoptotic protein Hid (Head Involution Defective) [39]–[41]. Hid over-expression in the developing eye (*GMR-hid*) triggers apoptotic cell death throughout the tissue leading to a small eye phenotype (Figure 4H) [42]. When dRET^WT^ was simultaneously expressed with Hid, the eye size increased significantly (Figure 4J and 4L), indicating that RET signalling could block Hid or its downstream effectors. The ability of dRET to suppress Hid-induced apoptosis depended on its known effectors Src and Ras: down-regulation of Ras85D or Src42A by RNA interference prevented dRET-mediated suppression of *GMR-hid* small eye phenotype (Figure S2B). In contrast, while dFAK expression by itself had no significant effects on the *GMR-hid* eye phenotype (Figure 4I), it did suppress dRET inhibitory effect (Figure 4K and 4L).

Taken together, our data suggest that dRET expression suppresses both ectopic (Hid-induced) and developmental cell death via its known effectors Src and Ras, and this effect can be suppressed by dFAK co-expression. Therefore, in the retina, the output of dRET overexpression in a *dFAK* wild type background—genetically defined here as a moderate RET/FAK ratio and expected to produce a moderate level of Ras/MAPK signalling— is the inhibition of cell death.

### High RET/FAK ratios drive ectopic differentiation

**Figure 5:**
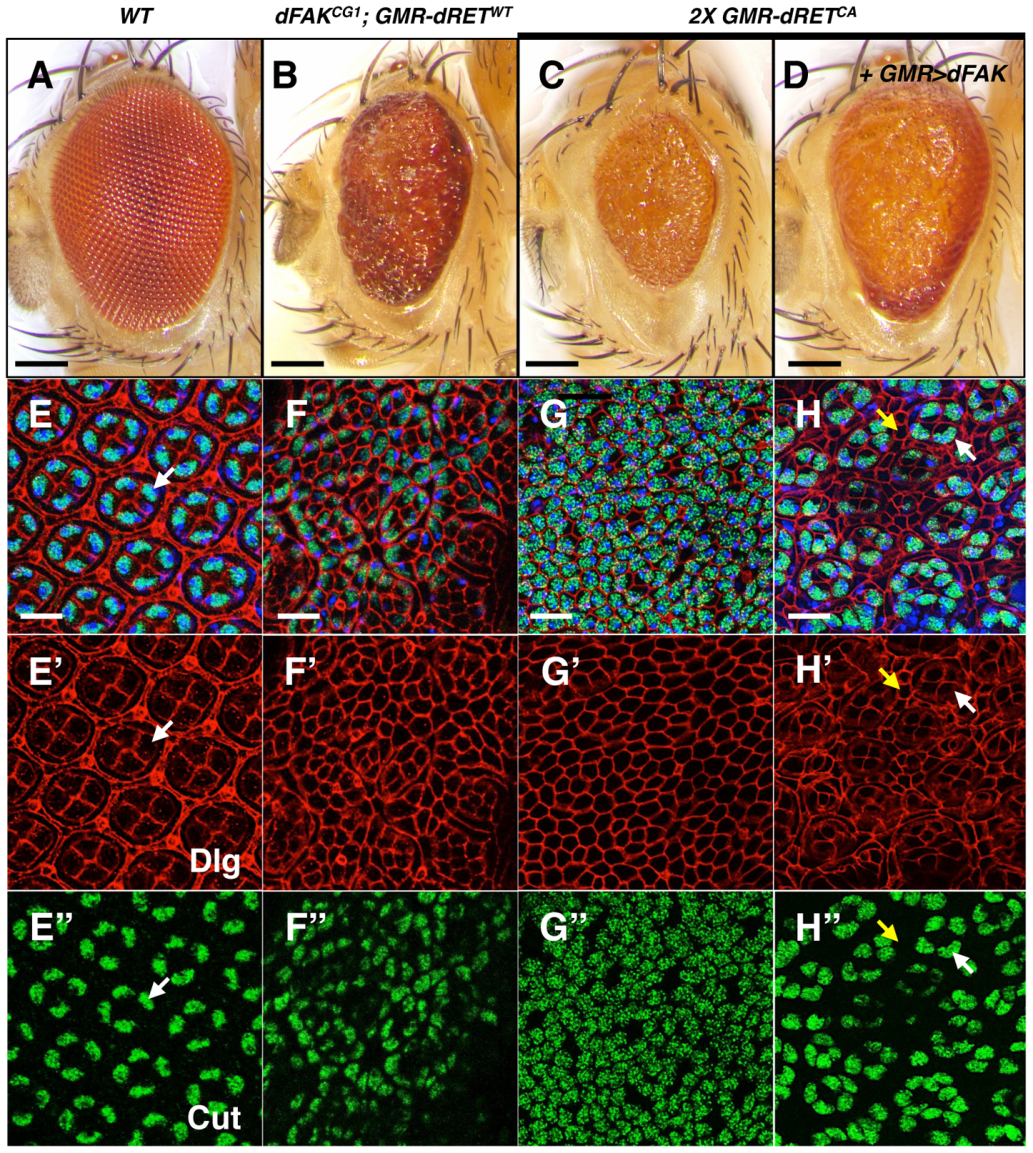
High relative levels between RET and FAK induce ectopic cone cell differentiation in the eye. We examined the cellular patterning of the pupal retinas in correspondence to the adult eye phenotypes shown in panels A–D. Scale bars, 100 µm. (E–H) Merged images of stainings for nuclei (DAPI, blue), Dlg (cell outlines, red) and Cut (cone cells, green), from retinas at 42 h APF. Bottom panels show Dlg (E′–H′) and Cut (E″–H″) immunostainings individually. (E) Note the symmetric hexagonal array, and four Cut^+^ cone cells per ommatidium (white arrows) in control retinas. (F and G) Note the change in cellular composition of these retinas with high RET/FAK ratios, primarily composed of Cut^+^ cone-like cells. (H) dFAK expression within a *2X GMR-dRET^CA^* background suppressed this phenotype (also see S1D); some normal four-cone cell clusters (white arrows) can be identified and interommatidial cells reappeared (yellow arrows). Scale bars, 10 µm.

While the output of moderate RET/FAK ratios resulted in suppression of cell death, the small eye phenotypes observed under conditions of higher RET/FAK ratios suggested different cell fate outcomes. We then further analysed two different experimental conditions expected to produce high relative levels between RET and FAK, namely (i) the expression of one copy of dRET^WT^ in a *dFAK* mutant background (*dFAK^CG1^; GMR-dRET^WT^*), and (ii) the expression of two copies of dRET^CA^ in a *dFAK* wild type background (*2X GMR-dRET^CA^*), as these displayed the roughest, reduced eye size phenotypes (Figure 5B and 5C, see also Figure 1J and O). *dFAK^CG1^; GMR-dRET^WT^* pupal retinas lacked the hexagonal array and identifiable ommatidial units (Figure 5F–F′). In this case, there were clusters of numerous cone-like cells. Some bristle cells and a few cells recognisable as primary pigment-like remained, but there were no detectable cells with the appearance of normal interommatidial cells (Figure 5F′). In order to confirm the identity of those cells, we stained for the transcription factor Cut, a well-known cone cell marker [43]. In control retinas, Cut localised constitutively to the nucleus of the four cone cells from each ommatidium (Figure 5E″) [43]. In contrast, in *dFAK^CG1^; GMR-dRET^WT^* retinas (Figure 5F″), the cell clusters were indeed made of numerous Cut-expressing cone-like cells. Thus, in these experimental conditions, dRET signalling drives ectopic differentiation into the cone cell fate.

We next tested whether the supernumerary cone cells could be a consequence of ectopic differentiation during the larval stage. Since photoreceptor cells induce cone cell differentiation, one possibility is that aberrant photoreceptor cell numbers trigger ectopic cone cell differentiation. We observed normal numbers of photoreceptors (i.e.; clusters of eight) in *dFAK^CG1^; GMR-dRET^WT^* eye discs, albeit some clusters had rotation defects (Figure S3A–C). We also observe ommatidial units with extranumerary cone cells at early stages of eye development (4 h APF; Figure S3E) that suggests these ectopic clusters might accumulate during development until a mesh of mostly cone cells is observed by 42 h APF.

Similarly, *2X GMR-dRET^CA^* pupal retinas displayed a mesh of cone-like cells as shown by Cut staining (Figure 5C and 5G–G″), where the few remaining cells displayed bristle cell morphology. The expression of dFAK within this context rescued the small eye size phenotype (Figure 5D; quantified in S1D). Most remarkably, it also reduced the number of ectopic cone cells and resulted in the re-appearance of normal ommatidial cores and surrounding interommatidial cells in the pupal retinas (Figure 5H–H″). Moreover, the down-regulation of Ras85D also prevented this ectopic differentiation, restoring the eye size and suppressing patterning defects (Figure S3D).

Together, these results indicate that in a genetically defined high RET/FAK ratio, expected to produce high Ras/MAPK signalling, most of non-neuronal eye cell types ectopically differentiate into cone cells.

### FAK impairs MAPK signalling downstream of RET

Next, in order to gain insights into the mechanism of dRET signalling inhibition, we assessed the role of dFAK in influencing dRET-signalling effectors.

dRET was reported to activate the PI3K/Akt pathway [33], therefore we assessed whether there was also an ectopic activation of *Drosophila* Akt1 in our experimental conditions. Overexpression of *Drosophila* Insulin receptor (dInR), an RTK known to signal primarily via the PI3K/Akt pathway [44], resulted in increased immunostaining for phosphorylated-Akt1 (pAkt) (Figure S4E and S4G); nevertheless, that was not the case after expression of dRET^CA^ (Figure S4C and S4G). This suggests that dRET signals independently of PI3K/Akt in the imaginal disc domains we utilized.

We showed above that Src and MAPK were activated upon RET expression (Figure 1D–E). dFAK overexpression on its own within the *ptc* domain of the wing disc also resulted in increased levels of pSrc (Figure S4I). However, the co-expression of dRET and dFAK did not affect notably the levels of pSrc (Figure S4K and S4L). These results indicate that dFAK does not suppress dRET signalling via Src.

**Figure 6:**
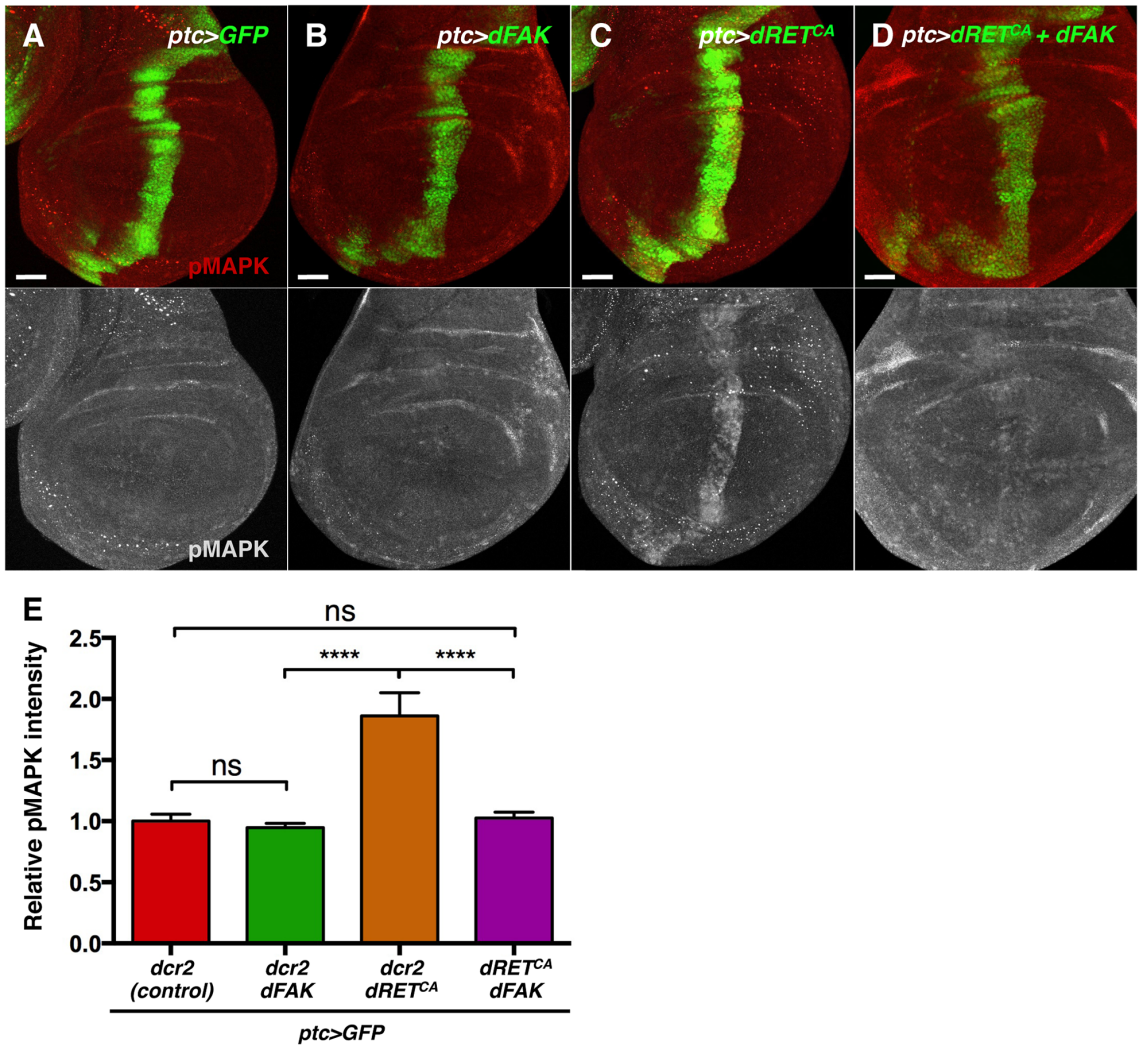
FAK inhibits RTK signalling by impairing Ras/MAPK pathway. (A–D) Phosphorylated (active) MAPK immunostainings from wing discs with the indicated genotypes. (A–B) When dFAK was expressed in the *ptc*-compartment (green), pMAPK staining was unchanged compared to GFP-only expressing cells. (C–D) dRET^CA^ expression increased pMAPK staining in the *ptc* domain but co-expression with dFAK suppressed this dRET^CA^-induced activation of MAPK. Scale bars, 50 µm. (E) Quantification of pMAPK immunostaining within the *ptc* stripe (see methods). Intensity of pMAPK signal is represented as relative values to the mean intensity of control tissues (A) (‘ns’: not statistically significant; **** = p<0.0001; n = 4–6 for each genotype).

Regarding MAPK, dFAK expression did not modulate MAPK phosphorylation when expressed by itself (*ptc>dFAK*) (Figure 6B) but did reduce MAPK phosphorylation within the dRET^CA^-overexpressing ptc compartment of the wing disc (Figure 6C–E). This indicates that dFAK is able to inhibit dRET signalling via the MAPK pathway.

Interestingly, the eye phenotypes mediated by activated isoforms of Ras and Raf, two components of the MAPK kinase pathway, were not suppressed by dFAK co-expression (Figure S5), suggesting that dFAK-mediated inhibition of dRET/MAPK signalling occurs upstream of Ras.

### FAK suppresses EGFR signalling

**Figure 7:**
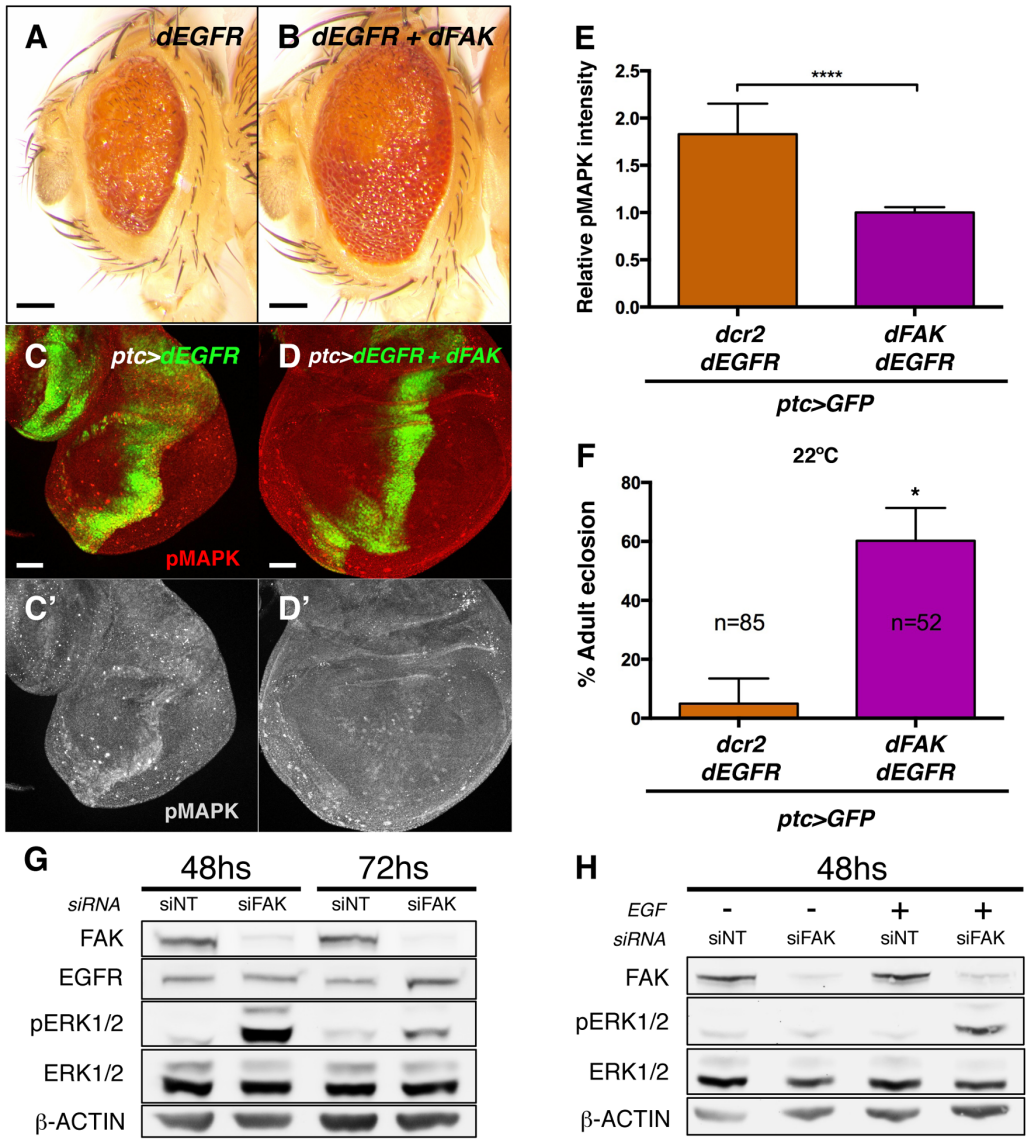
FAK suppression of RTK signalling is conserved. (A–B) Adult eyes images of animals expressing *Drosophila* EGFR (dEGFR) alone or in combination with dFAK. Note that dFAK expression suppressed the rough, small eye phenotype driven by dEGFR. Scale bar, 100 µm. (C–C′) Expression of dEGFR within the *ptc* domain resulted in increased MAPK phosphorylation, and co-expression of dFAK rescued the ectopic pMAPK staining within the *ptc* stripe (D–D′). Scale bar, 50 µm. (E) Quantification of pMAPK immunostaining within the *ptc* stripe (see methods). Intensity of pMAPK signal is represented as relative values to the mean intensity of control tissues (Figure 6A; **** = p<0.0001; n = 4–6 for each genotype). (F) Quantification of the penetrance on adult eclosion for the indicated genotypes. Note that dFAK co-expression significantly rescued the developmental lethality associated to *ptc*-driven dEGFR expression (* = p<0.05). (G) Western blots from protein extracts from MDA-MB-231 cells after 48 or 72 h transfection with FAK siRNA. FAK protein levels were effectively knocked down. While total levels of EGFR and ERK were not changed at 48 h, there was a marked upregulation in phosphorylated ERK1/2 upon FAK knockdown, which was more apparent at 48 h after siRNA transfection. Actin levels were probed as an additional loading control. (H) MDA-MB-231 cells were transfected with either non-targeting (siNT) or FAK-specific siRNA (siFAK) and serum starved prior to addition of EGF. Note that FAK knockdown resulted in increased phosphorylation of ERK1/2 in response to EGF treatment (30 µM, 15 minutes).

Next, we evaluated whether the ability of FAK to inhibit RTK/MAPK signalling was specific to RET. The epithelial growth factor receptor (EGFR) is known to bind to FAK in mammals [29], [45] and to activate MAPK in *Drosophila* [46]. Therefore, we took a similar approach and co-expressed the dEGFR with dFAK in the eye or in the *ptc* domains through the *GMR-gal4* and *ptc-gal4* drivers, respectively. As it happened with dRET, dEGFR also induced dFAK (Figure S6A) but not Akt1 activation (Figure S6B–B′). Co-expression with dFAK resulted in a significant rescue of the *GMR>dEGFR* phenotype (Figure 7A and 7B) and inhibition of MAPK activation (Figure 7C–E). Moreover, dFAK allowed a remarkable increase in survival of *ptc>dEGFR* flies (Figure 7F).

Mechanistically, dFAK seems to act in a similar fashion with both RTKs as the mutant isoforms of dFAK showed the same pattern of suppression when co-expressed within a *GMR>dEGFR* context: the auto-phosphorylation mutant was still capable of rescuing (Figure S6F), while the amino-terminal domain mutant failed to modify the phenotype of dEGFR overexpression (Figure S6E). Thus, the FERM domain of dFAK appears essential to initiate the negative regulation over RTKs.

This implies that dFAK not only suppressed dRET but can also suppress other RTKs, namely dEGFR.

### Phylogenetic conservation role for FAK downstream of RTK signalling

We next tested whether the negative role of FAK downstream of RTK signalling was evolutionary conserved. Since FAK inhibited dEGFR signalling, we utilized MDA-MB-231 cells, derived from a human breast adenocarcinoma, which express high levels of EGFR and FAK [47]–[50]. An efficient knockdown of the FAK protein was achieved using small interfering RNAs by 48 hours after transfection (Figure 7G and S7A). Remarkably, when cells were grown in presence of serum we observed an increase of ERK1/2 (MAPK) phosphorylation (pERK1/2) after FAK knockdown, while the total ERK1/2 and EGFR levels remained constant. ERK1/2 phosphorylation levels were also increased at 72 hs after transfection but at this later time point EGFR levels were mildly upregulated (Figure 7G and S7A).

When serum-starved FAK-siRNA MDA-MB-231 cells were treated with EGF in order to selectively stimulate EGFR receptor, the same dramatic increase of ERK (MAPK) activation was observed (Figure 7H and S7B). These results indicate that the suppressive role of FAK on RTK/MAPK signalling is evolutionary conserved through EGF/EGFR signalling in human breast cancer cells.

### FAK reduces the fraction of EGFR located at the cell surface

To gain mechanistic insights into how FAK suppresses RTK/MAPK signalling, we next examined the cellular distribution of EGFR in MDA-MB-231 cells. Previous work indicated that growth factor receptors regulate cell signalling differently depending on its localization at the plasma membrane or within internalized vesicles [51]–[53]. In most contexts, EGFR signals to MAPK preferentially when located at the cell surface [54], [55]. Since knockdown of FAK at 48 h did not affect total levels of EGFR, we hypothesised that a change of receptor subcellular localization could explain the enhanced ERK signalling.

**Figure 8:**
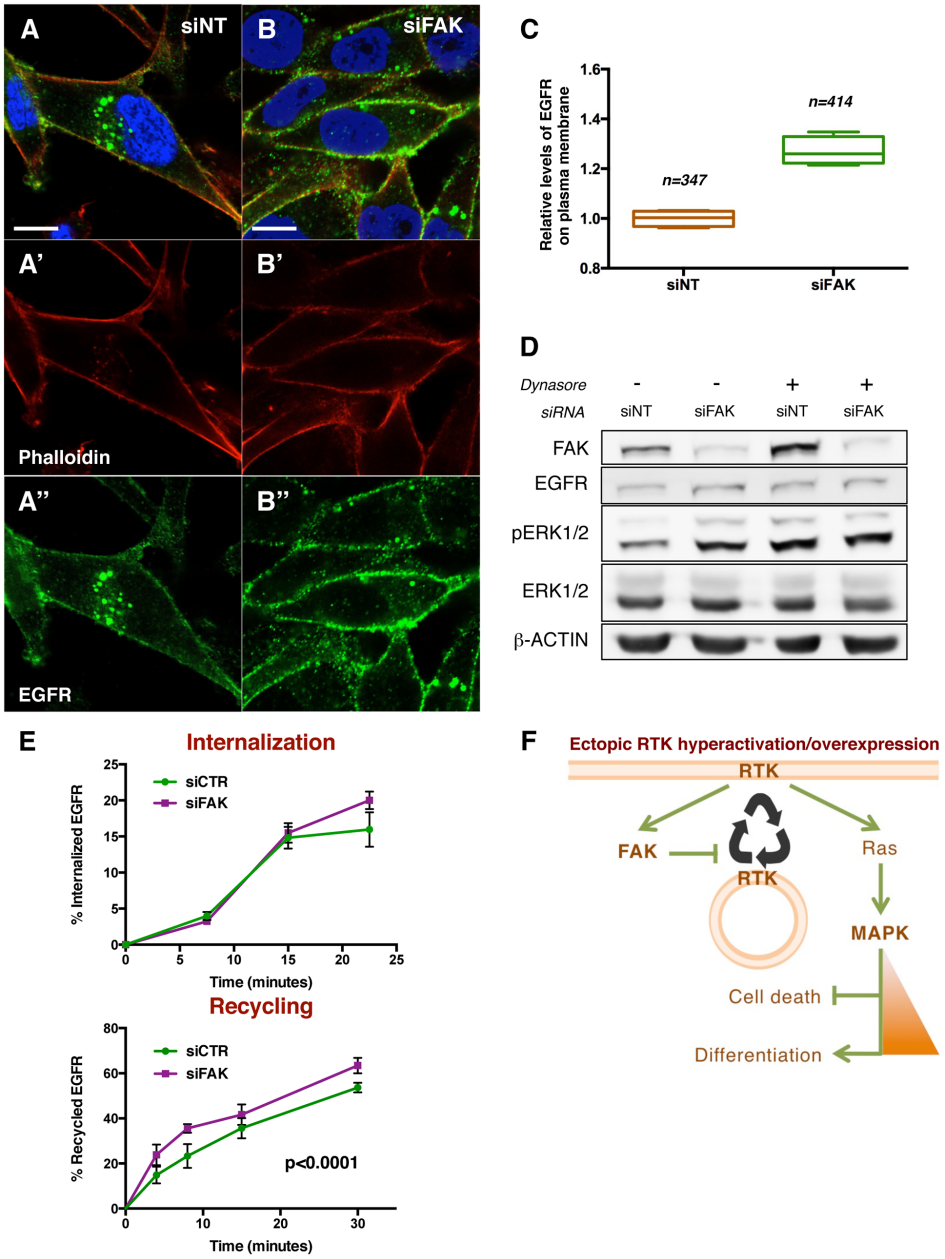
FAK decreases EGFR at the plasma membrane via reduced recycling. (A–B) MDA-MB-231 cells transfected with non-targeting (NT) siRNA or FAK siRNA were immunostained with anti-EGFR antibody (green, A″ and B″), Rhodamine-phalloidin (red, A′ and B′) and DAPI (blue). Note the differential localisation of EGFR; while in siNT cells the receptor is distributed in plasma membrane and internal vesicles, FAK downregulation leads to an increase of EGFR levels at the cellular membrane. Scale bar, 10 µm. (C) Quantification of relative EGFR membrane levels, values are expressed as relative levels of the receptor against the mean value of siNT cells; four confocal fields for each condition were analysed: n = 347 cells (siNT), and n = 414 cells (siFAK). p<0.0286 in a Mann-Whitney test. (D) MDA-MB-231 cells were transfected with either non-targeting (siNT) or FAK-specific siRNA (siFAK) and deprived of serum prior to addition of 80 µM Dynasore. siNT-transfected cells showed an increased pERK1/2 level in response to both Dynasore treatment (80 µM, 30 minutes) and FAK knockdown. Total levels of EGFR and ERK were not changed and actin levels were probed as an additional loading control. (E) The internalization of membrane EGFR (top panel) and recycling of internalised EGFR (bottom panel) were determined in MDA-MB-231 cells transfected with non-targeting siRNA (siCTR) or FAK siRNA (siFAK). Values are means ± Standard Deviation (SD) of two independent experiments with four to eight replicates of each time point per genotype. See materials and methods for more details. FAK knockdown did not affect receptor internalization but increased the recycling of the internalised EGFR pool. (F) A working model for the regulatory mechanism of FAK. Ectopic expression and/or hyperactivation of RTKs activate FAK and Ras among other signalling pathways. FAK mediates a negative regulation of receptor recycling; when FAK is reduced or absent, there are more RTKs molecules at the plasma membrane, thus enabling a higher flux of signalling through Ras/MAPK pathway. See the text for more details.

To assess whether FAK affects receptor subcellular localization, we performed immunofluorescence stainings for EGFR in control and FAK knockdown cells (Figure 8A and 8B). A 27% increase in the fraction of total EGFR located at the plasma membrane was observed in siFAK-treated cells, at the expense of the intracellular pool (Figure 8C). This suggested that the increase of EGFR at the cell surface could account for the increased ERK signalling in cells with reduced FAK levels. To further test this hypothesis, we experimentally increased the fraction of EGFR at the plasma membrane using the Dynamin GTPase inhibitor Dynasore, widely used to retain receptors at the cell surface [56]–[59]. This treatment phenocopied FAK knockdown as it led to increased pERK1/2 levels without affecting ERK1/2 or EGFR total levels (Figure 8D and S8).

Taken together, these results indicate that FAK disfavours localisation of EGFR at the plasma membrane thus reducing receptor signalling into the MAPK pathway.

### FAK reduces EGFR recycling to the cell surface

Finally, we asked how FAK might affect the receptor sub-cellular localisation. Receptors are normally internalised into the endosomal/lysosomal compartment, when they can be either degraded or spared from degradation and returned to the cell surface [60].

We performed pulse-chase biochemical assays [61] to quantify the rates of EGFR internalisation and recycling. We did not detect significant differences in the rate of EGFR internalization (Figure 8E). Instead, the recycling of the internal EGFR pool was significantly higher (36%) in cells with reduced FAK versus control cells (Figure 8E). These results correlate with the percentages of membrane EGFR observed in Figure 8A–C and hence indicate that FAK suppresses EGFR localisation at the plasma membrane by suppressing receptor recycling but not internalization.

## Discussion

This study provides evidence that RTK signalling can be moderated by FAK through the regulation of RTK recycling. This mechanism appears specific for the case of RTK over-expression/hyper-activation. We found that ectopic dRET and dEGFR signalling were able to activate dFAK and the MAPK pathway. Nevertheless, dFAK negatively regulated RTK-induced signalling to the MAPK pathway. The highly conserved FERM domain of dFAK appeared necessary for its functional suppression of *Drosophila* RTK signalling, which is consistent with the physical interactions of FAK and several RTKs described in mammals [28], [30], [62]. This negative feedback regulation indicates that the balance between RTK and FAK is what determines the intensity of MAPK pathway activity, which ultimately dictates cellular fate (Figure 8F).

We characterized cell fate outcomes in detail in the patterning eye anlage. In this tissue, it is known that different thresholds of MAPK pathway activation result in different outcomes: moderate levels of activation promote survival of cells during the wave of developmental apoptosis [63], while high levels of activation result in ectopic differentiation into photoreceptors [63] or the cone cell fate [64]. Correspondingly, we observed that experimental genetic manipulations expected to produce moderate RET/FAK ratios —such as the expression of one copy of wild type dRET in a *dFAK* wild type background— resulted in reduced developmental apoptosis and supernumerary interommatidial cells. On the other hand, genetically defined high RET/FAK ratios —such as the expression of one copy of wild type dRET in a *dFAK* mutant background, or the expression of two copies of constitutively activated dRET in a *dFAK* wild type background— resulted in ectopic differentiation into cone cells. Importantly, all dRET-driven phenotypes were suppressed by the co-overexpression of dFAK, which lowered RET/FAK ratios.

The initial characterization of the dRET-driven eye model identified several components of the Src and Ras/MAPK pathways [19]. These authors further observed that high levels of dRET signalling, achieved by the expression of two copies of activated RET, resulted in non-patterned retinas composed of identical cells with cuboidal morphology, which were proposed to be undifferentiated precursor cells. This led to the conclusion that high dRET signalling could block differentiation in this tissue. We report here that under similar experimental conditions, such cuboidal cells express the cone cell marker Cut. Thus, we conclude that high relative levels between dRET and dFAK result in a proportional activation of the MAPK pathway, which force differentiation into the cone cell fate.

Previous work reported that in *dFAK* mutant embryos, the activity of the MAPK pathway is normal during development [16]. This indicates that endogenous MAPK signalling does not normally became hyper-activated simply as a result of dFAK loss and is in sharp contrast to the case of over-expression of RET or EGFR as reported here. We postulate that in imaginal disc epithelia, dFAK constitutes a signalling “fuse” that can act in a negative feedback loop over RET and other RTKs, specifically in conditions of ectopic RTK activation or expression. Relevant to this, we recently reported that mammalian FAK can protect epithelial cells from deregulated RET or Src signalling; when FAK is absent, some epithelial cancer cells specifically respond by targeting these promiscuous oncogenic kinases for autophagic degradation [31], [65]. It therefore seems that a common feature of FAK regulatory function is to ‘buffer’ cells against potentially hazardous tyrosine kinases-mediated oncogenic signalling by promoting their internalization [31], [65].

In *Drosophila*, negative regulation of MAPK by dFAK has been previously observed in neuromuscular junction (NMJ) growth [16]; in fact, this was one of the few developmental defects detectable in *dFAK* mutant animals. Interestingly, it was suggested that this ‘non-canonical’ negative regulation of MAPK by dFAK was specific to the process of Integrin-dependent NMJ growth [16]. Importantly, our data imply instead that this is a more commonly used mechanism that occurs also in epithelial tissues, downstream of ectopic dRET and dEGFR signalling. Thus, the negative regulation of RTK/MAPK signalling by dFAK is more widespread across *Drosophila* tissues, and we show that this has important consequences for cell and tissue fate. Most importantly, we observed that the ability dFAK to restrain signalling through the MAPK pathway is evolutionary conserved in a human breast carcinoma cell line downstream of EGFR signalling. We demonstrate that this novel role of FAK relies on its ability to affect receptor sub-cellular localisation. RTKs normally reside at the plasma membrane or within internalised cytosolic vesicles. There is a constant transport of vesicles between these two pools, which allow cells to keep a healthy RTK signalling by degrading old receptor molecules or resetting their activity and sending them back to the cell surface. RTKs can activate a certain signalling pathway either from the plasma membrane or endocytic vesicles; here we showed that EGFR signals to Ras/MAPK pathway preferentially from the cell surface, and FAK is able to regulate its localisation by reducing recycling, while the internalization rates are unaffected. Thus, in a context where MAPK signalling is activated preferentially by the plasma membrane pool of EGFR [51]–[53], an increase of receptor located at the cell surface results in enhanced MAPK signalling.

We also present evidence that the N-terminal FERM domain of dFAK may be essential in the regulation of RTKs, while its kinase activity appears dispensable. These results highlighted the important regulatory roles of the FERM domain, likely by mediating interactions with RET and EGFR. Future studies should elucidate how FAK, presumably acting as a scaffold through its FERM domain, interacts with the recycling endosome machinery to control RTK recycling.

The attenuation of the MAPK signal transduction pathway by FAK is in stark contrast to the well-established role of FAK linking integrin engagement to the activation of Ras/MAPK [66]. Therefore, the regulation of MAPK by FAK may be context-dependent. It is worth noting that most previous studies reporting the activation of MAPK by FAK utilized immortalized cultured fibroblasts [66]; it is possible that FAK negative regulation of MAPK applies to epithelial cells *in situ* and acts downstream of RTKs but Integrins.

High expression or activation of FAK in a range of human carcinomas [49] and its role promoting migration and survival of malignant cells, make it an attractive therapeutic target. In fact, many small molecule inhibitors have been developed to target FAK kinase activity or its FERM domain, and clinical trials are in progress [67] [68]. However, in some tumors FAK downregulation has also been related with malignancy [69]–[72]. For instance, in glioblastoma, a malignancy that is associated with EGFR activation, there is an inverse correlation between pFAK and pMAPK [73], as predicted in our model.

Therefore, the signaling crosstalk between FAK and RTK/MAPK is complex and context-dependent [73], and a better understanding of such roles of FAK will be useful as FAK inhibitors move towards potential clinical use.

## Materials and Methods

### Drosophila stocks and culture

The following fly stocks were used: *w1118* and Canton-S as a wild-type reference; *GMR-gal4 and sev-gal4* as expression drivers in the eye [74]; *patched (ptc)-gal4* and *decapentaplegic (dpp)-gal4* as expression drivers in a stripe of cells in the wing and genital discs [34]; the *dFAK^CG1^* mutant line, *UAS-dFAK*, *UAS-dFAK^ΔN^* and *UAS-dFAK^Y430F^* lines were made by R. Palmer [13], [17]; *GMR-dRET^WT^, UAS-dRET^C695R(MEN2A)^* and *GMR-dRET^C695R(MEN2A)^* were a gift from R. Cagan (Mount Sinai Medical School, New York, USA) [19]; *dFAK^KG00304^*, *Ras85D^RNAi^*, *UAS-Ras85D^V12^*, *UAS-Raf^F179^, GMR-hid, and UAS-dInR lines* were obtained from the Bloomington Drosophila Stock Centre. *Src42A^RNAi^* line was obtained from the Vienna Drosophila RNAi Centre. *dFAK^5-SZ-3124^* was obtained from the Drosophila Genetic Resource Centre. The *UAS-dEGFR* line was obtained from Matthew Freeman [75]. All flies were cultured at 25°C on standard molasses diet unless otherwise stated. Please find in Text S1 the detailed genotypes of animals used in all figures.

### Overexpression using GAL4/UAS system

The *GMR-gal4* line drives expression of UAS-linked transgenes in differentiating and post-mitotic cells of the developing eye, posterior to the morphogenetic furrow; the *sevenless-gal4* line is active in R7 photoreceptor cells; the *patched-gal4 and decapentaplegic-gal4* lines drive expression of UAS-linked transgenes within compartments of cells in imaginal discs.

### Immunofluorescence assays

Eye and wing imaginal discs or pupal retinas were dissected at the indicated time points, in 1X PBS, fixed in 4% formaldehyde for 30 minutes at room temperature, and rinsed in PBST (PBS, 0.1% Triton X-100). Tissues were incubated in primary antibody at 4°C overnight. After PBST washing (3 times, 10 minutes each), tissues were incubated for 2 hours in secondary antibody. Tissues were rinsed in PBST and counterstained with DAPI (1 µg/ml, SIGMA) for 5 min at RT and then mounted in Vectashield. Primary antibodies used were anti-Armadillo (1∶3, DSHB) and anti-Cut (1∶50, DSHB), anti-phosphorylated (Y418)-Src (1∶100, Cell Signalling), anti-phosphorylated (Y397)-FAK (1∶100, Cell Signalling), anti-phosphorylated (T202/Y204)-MAPK (1∶200, Cell signalling), anti-Dlg (1∶50, DSHB), anti-Prospero (1∶30, DSHB), anti-Elav (1∶500, DSHB), anti-phosphorylated (S-473)-Akt1 (1∶100, Cell Signalling). Secondary antibodies were Alexa-conjugated 488, 594, or 633 (Molecular Probes). All preparations were analysed on a Zeiss 710 upright confocal microscope and images were processed with Fiji (ImageJ) program (NIH).

### Quantification of immunofluorescence stainings

In order to quantify the intensity of immunostainings within the *ptc* compartment of the wing discs, sum projections were created from the original multi-slice files. Two identical areas were defined, within the *ptc* domain (A1) and posterior to it (A2). The ‘mean grayscale value’ of each area was measured with Image J, and the ratio between A1 and A2 was taken as the normalised intensity of signal.

### TUNEL (Terminal deoxynucleotidyl transferase (TdT)-mediated dUTP Nick End Labelling)

Eye discs or retinas were dissected and fixed in 4% formaldehyde in PBT for 20 min at RT. Samples were permeabilized in 100 mM Sodium Citrate in PBST (PBS+0.1% Triton X-100) at 65°C for 30 min followed by the addition of TUNEL reagent according to the manufacturer's instructions (In Situ Cell Death Detection Kit, Roche) and incubated at 37°C for 2 h on a rotating platform.

### Light microscopy

Adult eye and male genitalia images were taken with a Leica M205 FA stereomicroscope equipped with Montage software. Wing blades images were taken with an Olympus BX51 FL Microscope.

### RNA quantification

Total RNA was extracted from 10–15 heads or whole bodies per biological replicate using RNeasy kit (Qiagen) and converted into cDNA using the High-Capacity cDNA Reverse Transcription Kit (Applied Biosystems). MAXIMA SYBR Green Master Mix (Fermentas) was used for quantitative (q) PCR. Data from three biological replicates were analyzed using Applied Biosystems 7500 software version 2.0 and GraphPad Prism 6 software. Data are presented as the mean fold change relative to wild type with standard deviations. Primers are listed in Text S1.

### Statistical analysis

To statistically analyse eye size measurements, adult eclosion and immunofluorescence signal we used Student's unpaired t-test to compare two groups of data or One-way ANOVA followed by Bonferroni's or Tukey's post-test corrections to compare more than two groups of data. Error bars are standard deviation in all plots.

### Cell culture and transfection

MDA-MB-231 cells were cultured in Dulbecco's modified Eagle medium (DMEM) supplemented with 2 mM glutamine and 10% FBS (Fetal Bovine Serum) at 37°C in a humidified 5% CO_2_ atmosphere. siGENOME non-targeting (NT) siRNA pool (D-001206-13-05) and Smartpool siRNAs targeting FAK (L-003164-00) were obtained from Dharmacon, 10 ul of the 20 uM stock was used in each transfection. Non-targeting and FAK siRNAs were transfected into cells using Nucleofector Technology (Nucleofector Solution V, program X-013; Lonza) and Nucleofector II, Amaxa Biosystems.

### Human cell imaging and analysis

MDA-MB-231 transfected cells were washed in ice cold PBS, fixed in 4% paraformaldehyde for 10 minutes at room temperature (RT), and permeabilised during 5 minutes in PBS+0.2% Triton X-100. Then, cells were blocked in 1% BSA/PBS solution for 30 minutes and incubated with primary antibody overnight. Secondary antibody was added to cells for 1 hour at RT and then cells were washed, incubated with FITC-Phalloidin for 10 minutes and finally mounted in Vectashield with DAPI.

Imaging was done with an inverted confocal microscope (FluoView FV1000; Olympus) with FluoView software (Olympus) and processed with Fiji (ImageJ) software (National Institute of Health). Immunofluorescence intensity values of EGFR were obtained by creating a mask of the cell outline, defining a threshold and measuring fluorescent intensity. Data analysis and Mann-Whitney statistical tests were performed and plotted in GraphPad Prism 6 software.

### Western blotting

Assays were set up 48 or 72 hours after transfection. EGF treatment (30 uM, 15 minutes - Millipore) was performed on serum-starved cells, whereas all other experiments were conducted in the presence of serum. Each assay was independently repeated three times with similar results; the blots with the most equalized loading as judged by β-actin labelling are shown.

Cells were washed in PBS (Phosphate buffered saline) and lysed in lysis buffer (2% SDS, 100 mM Tris-HCl, pH 7.4). Cell protein extracts were incubated at 95°C for 5 minutes, sonicated and clarified by centrifugation at 10,000 *g* for 15 min. Protein concentration was determined by Bradford method [76]. Proteins were resolved by SDS-PAGE and analysed by Western blotting. The following antibodies were used for immunoblotting: anti-β-actin (1∶10000, Abcam), anti-FAK (1∶1000, C-20, Santa Cruz Biotechnology), anti-phosphorylated (T202/Y204)-MAPK (pERK1/2) (1∶200, Cell signalling), anti-ERK1/2 (1∶10000, Promega), anti-EGFR (1∶2000, BD Transduction Laboratories). The Nitrocellulose membranes were incubated with secondary antibodies IRDye 680RD anti-mouse (1∶10000, LI-COR) and IRDye 800CW anti-rabbit (1∶10000, LI-COR) imaged with Odyssey Imager (LI-COR Biosciences) and analysed with Image Studio Lite software.

### Receptor internalization and recycling assays

EGFR internalization and recycling assays were performed as described previously for Integrins [61] [77], but without re-feeding or serum-starvation. For internalization assays: MDA-MD-231 cells were transferred to ice, washed twice in cold PBS, and their surface proteins labelled with 0.13 mg/ml NHS-SS-Biotin in PBS at 4°C for 1 h. Labelled cells were washed in cold PBS and transferred to serum-containing DMEM at 37°C for the indicated times to allow internalization. After internalization, dishes were rapidly transferred to ice and washed. Biotin remaining at cell surface was removed by incubation with 20 mM MesNa (sodium 2-mercaptoethanesulphonate), 50 mM Tris pH 8.6, 100 mM NaCl for 60 min at 4°C. MesNa was quenched by addition of 20 mM iodoacetamide for 20 min. Then, cells were washed and lysed in 200 mM NaCl, 75 mM Tris, 15 mM NaF, 1.5 mM Na_3_VO_4_, 7.5 mM EDTA, 7.5 mM EGTA, 1.5% Triton X-100, 0.75% IGEPAL CA-630, 50 µg/ml leupeptin, 50 µg/ml aprotinin, and 1 mM 4-(2-aminoethyl) benzynesulphonyl fluoride. Lysates were clarified by centrifugation at 10,000 *g* for 10 min, and biotinylated EGFR was determined by ELISA. Briefly, 96-well plates were coated overnight with monoclonal mouse anti–EGFR (clone EGFR.1; BD Biosciences) at 5 µg/ml at 4C° in 0.05 M Na_2_CO_3_ pH 9.6, and then blocked in PBS, 0.05% Tween 20, 5% BSA for 1 h at room temperature. EGF Receptors were captured by overnight incubation of 50 µl cell lysate at 4°C. After extensive washing with PBS-T to remove unbound material, wells were incubated with streptavidin-conjugated horseradish peroxidase for 1 h at 4°C. After further washing, biotinylated EGFR molecules were detected by chromogenic reaction with orthophenylenediamine.

For recycling assays, after surface labelling with Biotin, cells were transferred to serum-containing DMEM and incubated at 37°C for 30 min to allow internalization. Then, cells were returned to ice and washed in ice-cold PBS, and biotin was removed from cell surface proteins by reduction with MesNa. The internalized fraction was then chased by returning cells to 37°C in serum-containing DMEM. After recycling, cells were returned to ice, and biotin was removed from recycled proteins at the cell surface by a second reduction with MesNa. Biotinylated EGFR molecules were then determined by capture ELISA as described above. Two independent recycling experiments were performed with 4 to 8 independent measures of every time point and genotype. Two-way ANOVA with Sidak's multiple comparison test was performed to obtain statistical significance between time points.

## Supporting Information

Figure S1 Different *dFAK* alleles enhance eye roughness caused by dRET expression. (A) Adult eye images of the three *dFAK* mutant lines used in this study. Normal ommatidia patterning was observed. Scale bars, 100 µm. *dFAK^CG1^* is an amorphic allele of *dFAK* consisting of a deletion that removes the first 1263 base pairs of the coding sequence, corresponding to the first 421 amino acids of dFAK; please note this allele is in a *white* background [17]. (B) *dFAK^KG00304^* and *dFAK^5-SZ-3124^* are hypomorphic lines that bear two different P-element insertions in the same position of the gene, which resides within the 5′-unstranslated region (UTR) of the mRNA. *dFAK* mRNA levels from whole animal RNA extract were assessed by quantitative PCR (qPCR) using two pairs of primers: Pair 1 (1F/1R) flanks the P-element insertion site; Pair 2 (2F/2R) amplifies a region within the N-terminal domain spanning the amino acid residues 120 and 188. Note all three alleles result in very low or undetectable expression of the gene product. (C) Effect of independent *dFAK* mutant allelic combinations over dRET^WT^-driven rough phenotype. The three different *dFAK* mutant lines were combined to produce trans-heterozygous *dFAK* mutants: *dFAK^CG1^/dFAK^KG00304^; GMR-dRET^WT^* and *dFAK^CG1^/dFAK^5-SZ-3124^; GMR-dRET^WT^*, which showed phenotype similar to *dFAK^CG1^/dFAK^CG1^; GMR-RET^WT^* shown in Figure 1J. (D) Eye size quantification of the indicated genotypes, corresponding to Figure 1G, 1H, 1J, 1K, 1O and 1P. Eye size is represented as the relative value to the *wild type* mean (**** = p<0.0001; ** = p<0.01; ‘ns’: not statistically significant; n = 8–10 for each genotype).
(PDF)

Figure S2 Src kinase and Ras act downstream of RET. (A) *Src42A^RNAi^* or *Ras85D^RNAi^* expression in the eye with GMR-gal4 did not affect the adult eye pattern but suppressed *dRET^WT^*–induced rough eye phenotype. (B) *Src42A^RNAi^* and *Ras85D^RNAi^* also reduced the dRET^WT^-dependent suppression of Hid-induced apoptosis, proving this anti-apoptotic role of dRET was dependent on its effectors Src and Ras. (C) Expression of an autophosphorylation-site point-mutant of dFAK (dFAK^Y430F^) produces no defects in ommatidia patterning of the adult eye while suppressed the severe mis-patterning caused after expression of dRET^WT^ within a *dFAK* heterozygous background, further suggesting that the kinase activity of dFAK is not essential in this effect. Scale bars, 100 µm.
(PDF)

Figure S3 High RET/FAK ratios drive ectopic differentiation into cone cells but not photoreceptors. (A) Immunofluorescence stainings for the pan-neuronal marker Elav revealed that photoreceptor differentiation was not altered in *GMR-dRET^WT^* or *dFAK^CG1^; GMR-dRET^WT^* eye discs. (B) Staining for the R7-photoreceptor marker, Prospero, at later stages of eye development (42 h APF) showed one single R7 photoreceptor nuclei per cluster in all genotypes. Circles indicate bristle cell nuclei, which also express Prospero. Normally, these bristle and R7 nuclei are in different focal planes but appear together due to misfolding in *dFAK^CG1^; GMR-dRET^WT^* retinas. (C) Armadillo staining further demonstrated the normal clusters of photoreceptor cells. All the clusters showed seven photoreceptor cells although planar polarity rotation problems were observed in *dFAK^CG1^; GMR-dRET^WT^* retinas. (D) *Ras85D^RNAi^* expression suppressed the severely mis-patterned and small eye phenotype of *dFAK^CG1^; GMR-dRET^WT^* and 2X *GMR-dRET^CA^* flies (compare to Figure 1J and O, and 5B–C). This suggests that Ras/MAPK signalling is the main driving force of ectopic cone cell differentiation, which results in severe miss patterning. (E) Cut staining of early pupa retinas (4 h APF) for the indicated genotypes. Panels on the right show high magnification images. Note the presence of clusters with supernumerary cone cells in *dFAK^CG1^; GMR-dRET^WT^* retinas.
(PDF)

Figure S4 FAK does not regulate Src or AKT activation downstream of RTKs. (A–F) Phosphorylated Akt immunostainings, as proxy for its activation, in wing discs with the indicated genotypes. Note Akt phosphorylation was not activated by dRET^CA^ or dFAK expressing tissue, or simultaneous expression of both proteins in the *ptc* stripe of wing discs. In contrast, over-expression of *Drosophila* Insulin Receptor (dInR) did increase pAkt staining within the *ptc* stripe, but this was not reduced by dFAK co-expression. (H–K) Independent expression of dFAK and dRET^CA^ caused a significant activation of Src kinase in the *ptc* compartment of the wing disc, which was unchanged when both proteins were simultaneously expressed (L). (G and L) Quantification of pAkt and pSrc immunostaining, respectively, within the *ptc* stripe (see methods). Intensity of signal is represented as relative values to the mean intensity of control tissues overexpressing *GFP* and *dicer2* (A and H, respectively) (* = p<0.05; ** = p<0.01; ‘ns’: not statistically significant; n = 4–6 for each genotype). Scale bars, 50 µm.
(PDF)

Figure S5 FAK does not suppress effects of RTK/MAPK intermediaries Ras and Raf. The sevenless promoter driver *sev-gal4* was used to map the suppression of dFAK in the RET/MAPK pathway (*GMR-gal4* resulted in pupa lethality when driving *UAS-Ras85D^V12^* and *UAS-Raf^F179^*). dRET^CA^-induced patterning defects were suppressed by dFAK co-expression, while the effects produced by the expression of activated isoforms of Ras (Ras^V12^) or Raf (Raf^F179^) were not suppressed; moreover, patches of non-pigmented ommatidia appeared when dFAK was co-expressed, implying that dFAK suppresses dRET signalling upstream of Ras in the MAPK pathway.
(PDF)

Figure S6 FAK suppressed the RTK dEGFR. (A) Immunostaining assays showed increase phosphorylation of dFAK upon dEGFR expression in the *ptc* stripe of the wing disc. (B) dEGFR did not activate Akt1 phosphorylation significantly in the *ptc* stripe; dFAK co-expression made no difference either, as assessed by quantification of immunostaining signal (B′). Scale bar, 50 µm. Control genotypes were shown in Figure S4A–B. (C–F) Eye micrographs show the effects of different dFAK mutant isoforms on the dEGFR-overexpression phenotype. Note that similar to the case of dRET^CA^ (Figure 3), the N-terminus domain mutant (dFAK^ΔN^) did not affect dEGFR reduced eye size, while a full-length dFAK and a kinase mutant dFAK isoform (dFAK^Y430F^) did suppress this phenotype. Scale bars, 100 µm. (G) Quantification of eye sizes from the different genotypes, expressed as relative values to the mean value of control panel (C) (‘ns’: not statistically significant; *** = p<0.001; **** = *p*<0.0001; n = 8–10 for each genotype).
(PDF)

Figure S7 FAK negative regulation of EGFR is conserved in MDA-MB-231 human breast adenocarcinoma cells. Expansions of Figure 7G (A) and 7H (B), which shows the un-cropped western blotting images for each antibody labelling. Molecular weight markers (MM) are shown in either the first or last lanes, and the original multicolour Li-Cor scanned images are shown in the left panels. Expected molecular weights of proteins are: EGFR (175 kDa); FAK (125 kDa); ERK1/2 (44/42 kDa); β-actin (42 kDa).
(PDF)

Figure S8 Inhibition of dynamin-dependent internalisation retains EGFR at the plasma membrane. Expansion of Figure 8D showing the un-cropped western blotting images for each antibody labelling. Molecular weight markers (MM) are shown in the first lanes, and the original multicolour Li-Cor scanned images are shown in the left panels. Expected molecular weights of proteins are: EGFR (175 kDa); FAK (125 kDa); ERK1/2 (44/42 kDa); β-actin (42 kDa). MDA-MB-231 cells were transfected with either non-targeting (siNT) or FAK-specific siRNA (siFAK) and serum starved prior to addition of 80 uM Dynasore hydrate (Sigma Aldrich). Note that siNT-transfected cells showed an increased phosphorylation of ERK1/2 in response to Dynasore treatment (80 µM, 30 minutes).
(PDF)

Text S1 This text file provides a list of the full genotypes used in all figures, as well as a list of primer sequences used for PCR.
(DOCX)
