# Supplementary figures and images for: FAK Acts as a Suppressor of RTK-MAP Kinase Signalling in Drosophila melanogaster Epithelia and Human Cancer Cells
Source: PLoS Genet. 2014 Mar 27;10(3):e1004262. doi: 10.1371/journal.pgen.1004262 (PMC3967952; doi:10.1371/journal.pgen.1004262)

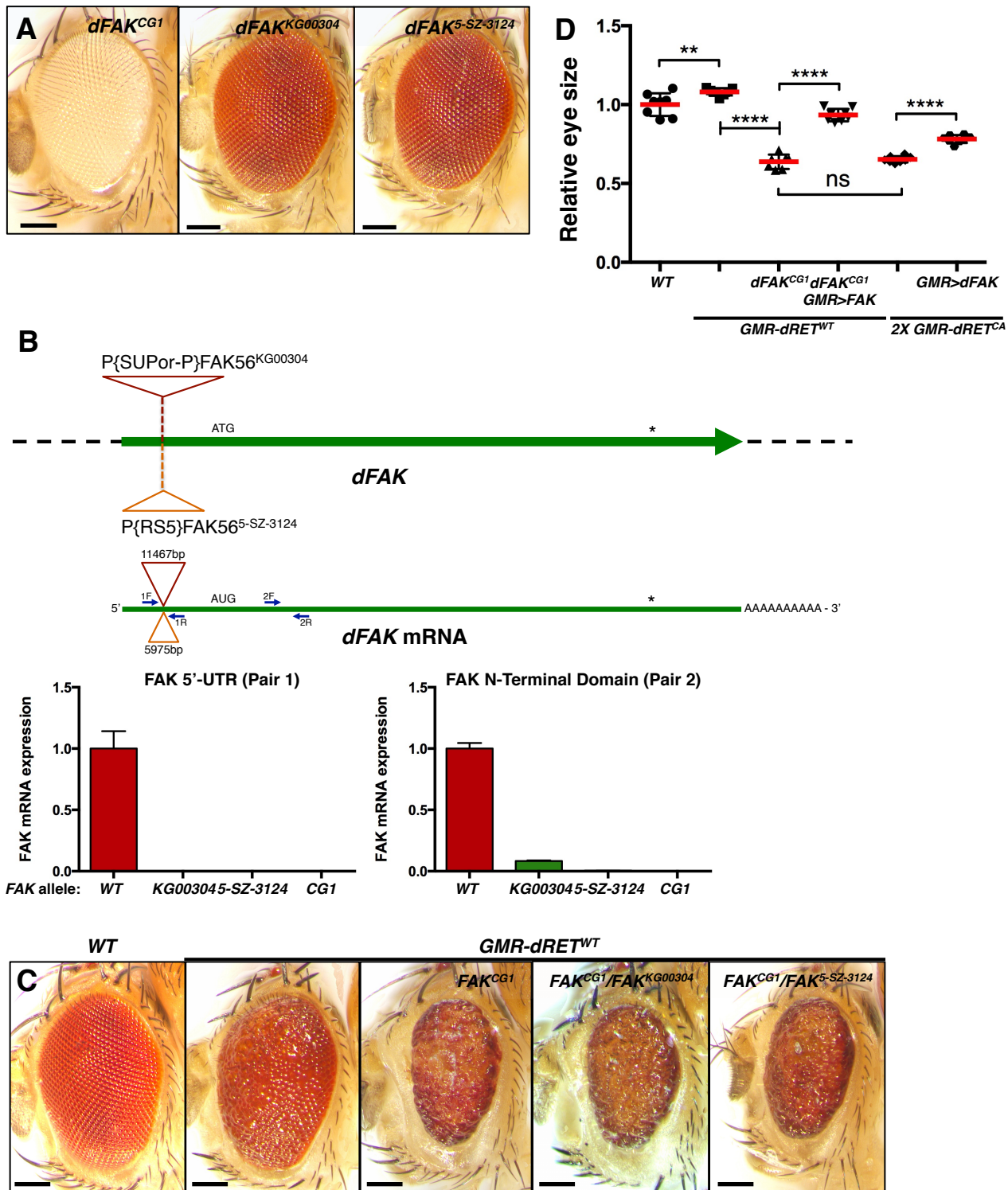

Supplement: Figure S1 — Different dFAK alleles enhance eye roughness caused by dRET expression. (A) Adult eye images of the three dFAK mutant lines used in this study. Normal ommatidia patterning was observed. Scale bars, 100 µm. dFAKCG1 is an amorphic allele of dFAK consisting of a deletion that removes the first 1263 base pairs of the coding sequence, corresponding to the first 421 amino acids of dFAK; please note this allele is in a white background [17]. (B) dFAKKG00304 and dFAK5-SZ-3124 are hypomorphic lines that bear two different P-element insertions in the same position of the gene, which resides within the 5′-unstranslated region (UTR) of the mRNA. dFAK mRNA levels from whole animal RNA extract were assessed by quantitative PCR (qPCR) using two pairs of primers: Pair 1 (1F/1R) flanks the P-element insertion site; Pair 2 (2F/2R) amplifies a region within the N-terminal domain spanning the amino acid residues 120 and 188. Note all three alleles result in very low or undetectable expression of the gene product. (C) Effect of independent dFAK mutant allelic combinations over dRETWT-driven rough phenotype. The three different dFAK mutant lines were combined to produce trans-heterozygous dFAK mutants: dFAKCG1/dFAKKG00304; GMR-dRETWT and dFAKCG1/dFAK5-SZ-3124; GMR-dRETWT, which showed phenotype similar to dFAKCG1/dFAKCG1; GMR-RETWT shown in Figure 1J. (D) Eye size quantification of the indicated genotypes, corresponding to Figure 1G, 1H, 1J, 1K, 1O and 1P. Eye size is represented as the relative value to the wild type mean (**** = p<0.0001; ** = p<0.01; ‘ns’: not statistically significant; n = 8–10 for each genotype). (PDF) [file pgen.1004262.s001.pdf]

**A**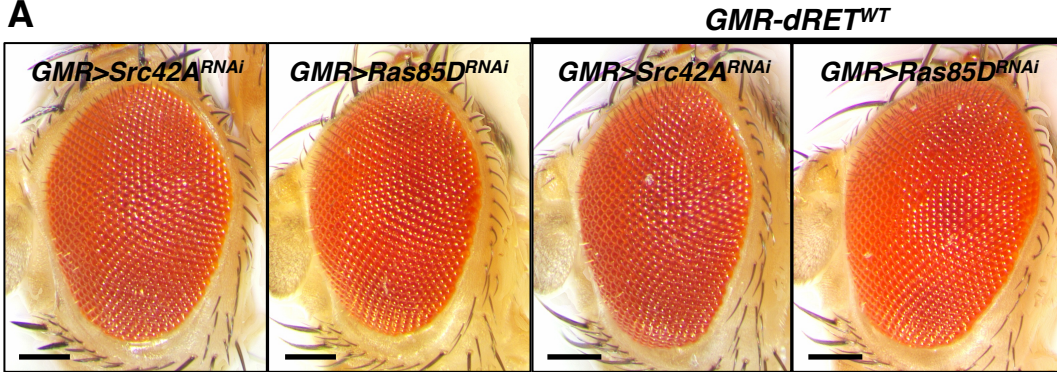**B**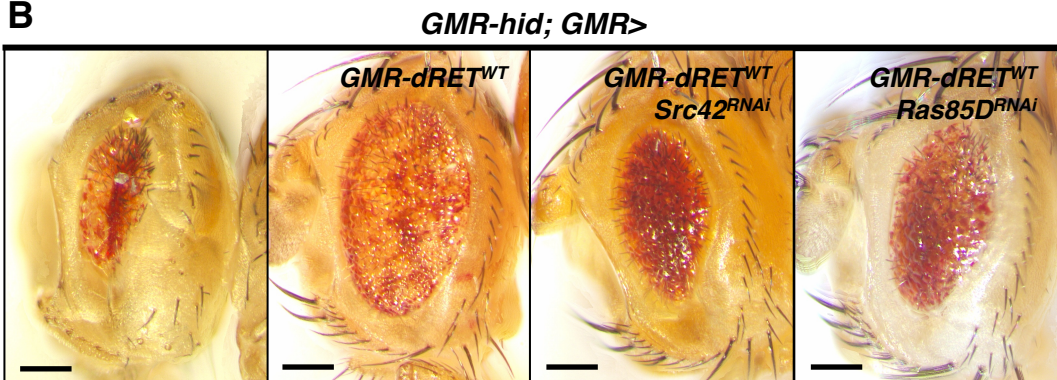**C**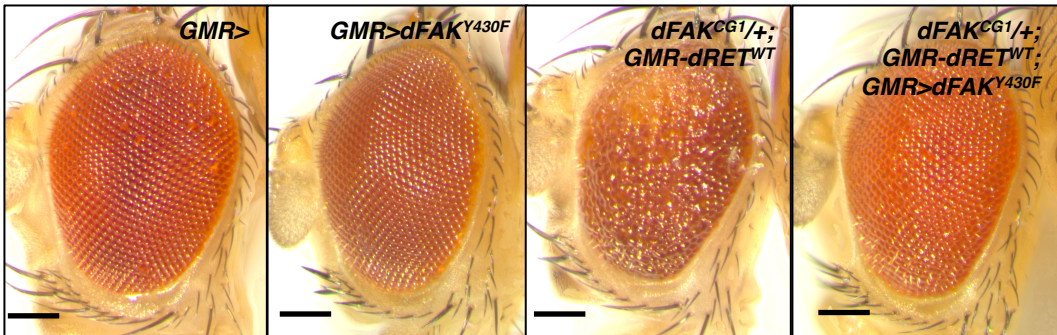

Supplement: Figure S2 — Src kinase and Ras act downstream of RET. (A) Src42ARNAi or Ras85DRNAi expression in the eye with GMR-gal4 did not affect the adult eye pattern but suppressed dRETWT–induced rough eye phenotype. (B) Src42ARNAi and Ras85DRNAi also reduced the dRETWT-dependent suppression of Hid-induced apoptosis, proving this anti-apoptotic role of dRET was dependent on its effectors Src and Ras. (C) Expression of an autophosphorylation-site point-mutant of dFAK (dFAKY430F) produces no defects in ommatidia patterning of the adult eye while suppressed the severe mis-patterning caused after expression of dRETWT within a dFAK heterozygous background, further suggesting that the kinase activity of dFAK is not essential in this effect. Scale bars, 100 µm. (PDF) [file pgen.1004262.s002.pdf]

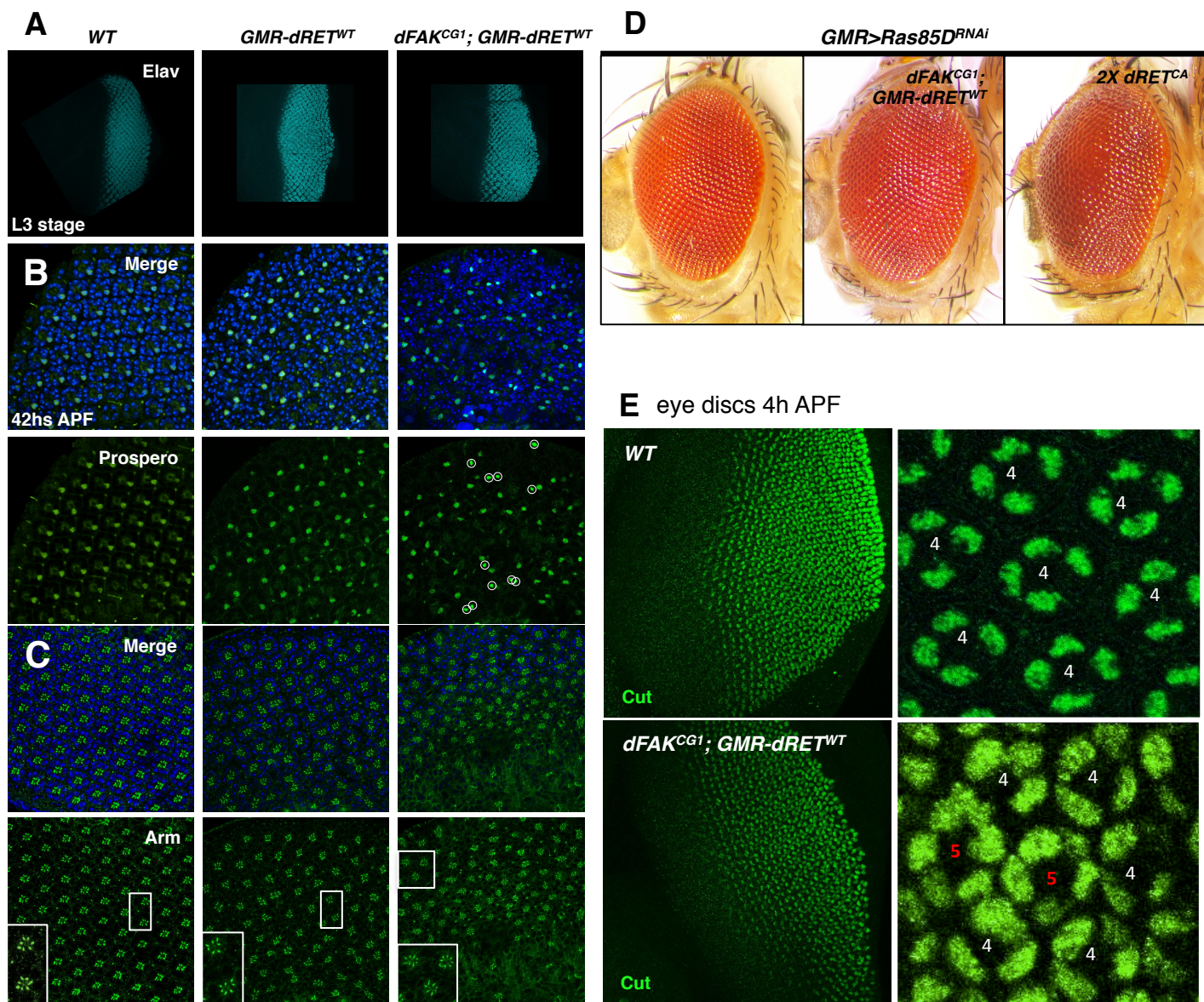

Supplement: Figure S3 — High RET/FAK ratios drive ectopic differentiation into cone cells but not photoreceptors. (A) Immunofluorescence stainings for the pan-neuronal marker Elav revealed that photoreceptor differentiation was not altered in GMR-dRETWT or dFAKCG1; GMR-dRETWT eye discs. (B) Staining for the R7-photoreceptor marker, Prospero, at later stages of eye development (42 h APF) showed one single R7 photoreceptor nuclei per cluster in all genotypes. Circles indicate bristle cell nuclei, which also express Prospero. Normally, these bristle and R7 nuclei are in different focal planes but appear together due to misfolding in dFAKCG1; GMR-dRETWT retinas. (C) Armadillo staining further demonstrated the normal clusters of photoreceptor cells. All the clusters showed seven photoreceptor cells although planar polarity rotation problems were observed in dFAKCG1; GMR-dRETWT retinas. (D) Ras85DRNAi expression suppressed the severely mis-patterned and small eye phenotype of dFAKCG1; GMR-dRETWT and 2X GMR-dRETCA flies (compare to Figure 1J and O, and 5B–C). This suggests that Ras/MAPK signalling is the main driving force of ectopic cone cell differentiation, which results in severe miss patterning. (E) Cut staining of early pupa retinas (4 h APF) for the indicated genotypes. Panels on the right show high magnification images. Note the presence of clusters with supernumerary cone cells in dFAKCG1; GMR-dRETWT retinas. (PDF) [file pgen.1004262.s003.pdf]

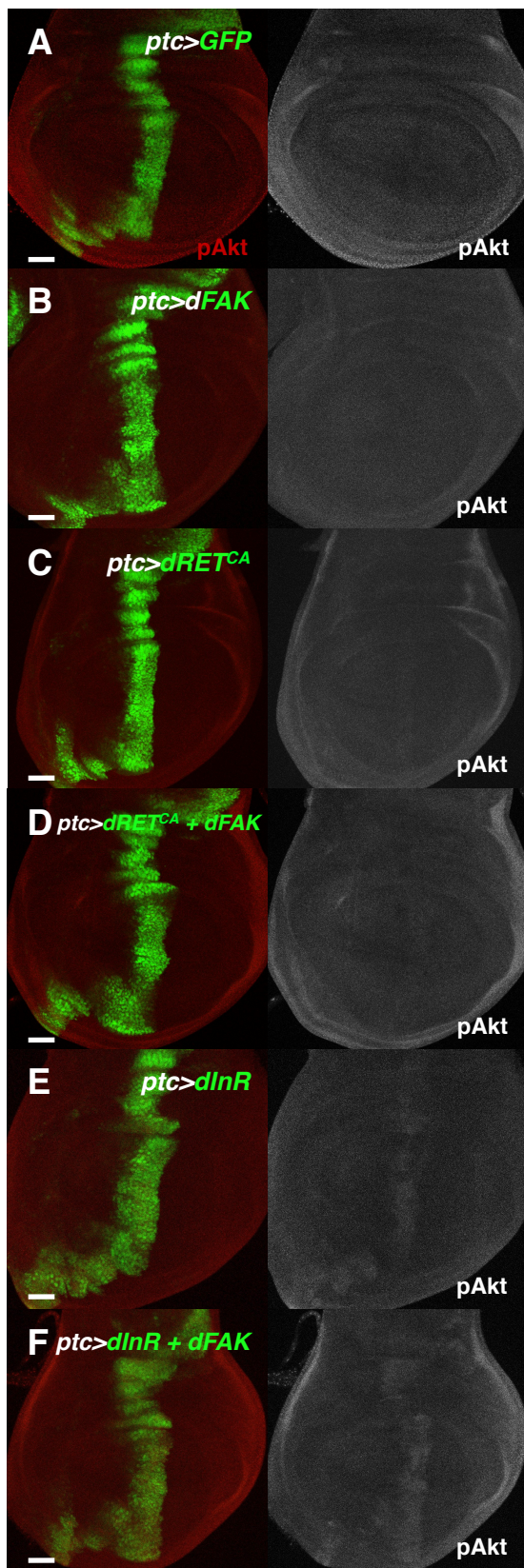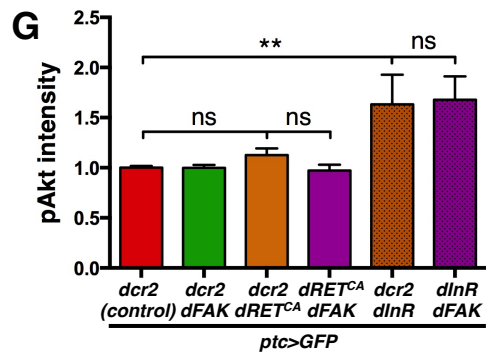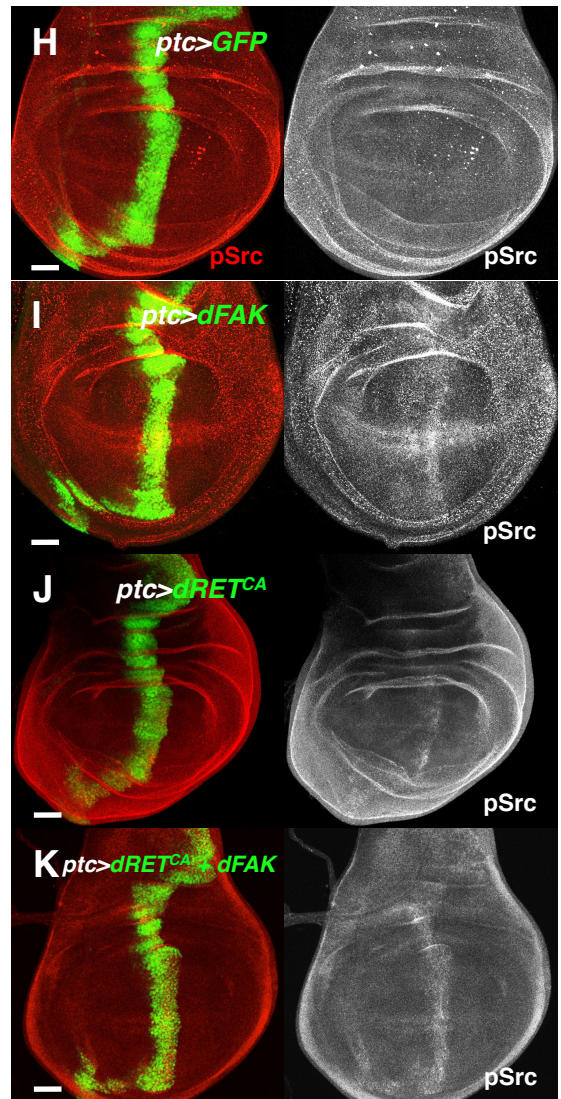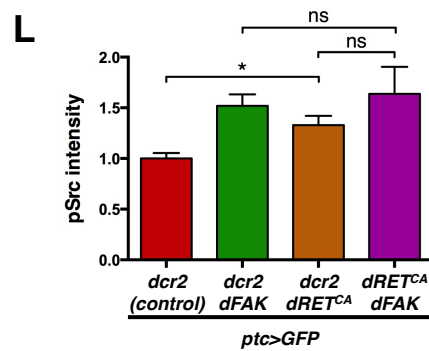

Supplement: Figure S4 — FAK does not regulate Src or AKT activation downstream of RTKs. (A–F) Phosphorylated Akt immunostainings, as proxy for its activation, in wing discs with the indicated genotypes. Note Akt phosphorylation was not activated by dRETCA or dFAK expressing tissue, or simultaneous expression of both proteins in the ptc stripe of wing discs. In contrast, over-expression of Drosophila Insulin Receptor (dInR) did increase pAkt staining within the ptc stripe, but this was not reduced by dFAK co-expression. (H–K) Independent expression of dFAK and dRETCA caused a significant activation of Src kinase in the ptc compartment of the wing disc, which was unchanged when both proteins were simultaneously expressed (L). (G and L) Quantification of pAkt and pSrc immunostaining, respectively, within the ptc stripe (see methods). Intensity of signal is represented as relative values to the mean intensity of control tissues overexpressing GFP and dicer2 (A and H, respectively) (* = p<0.05; ** = p<0.01; ‘ns’: not statistically significant; n = 4–6 for each genotype). Scale bars, 50 µm. (PDF) [file pgen.1004262.s004.pdf]

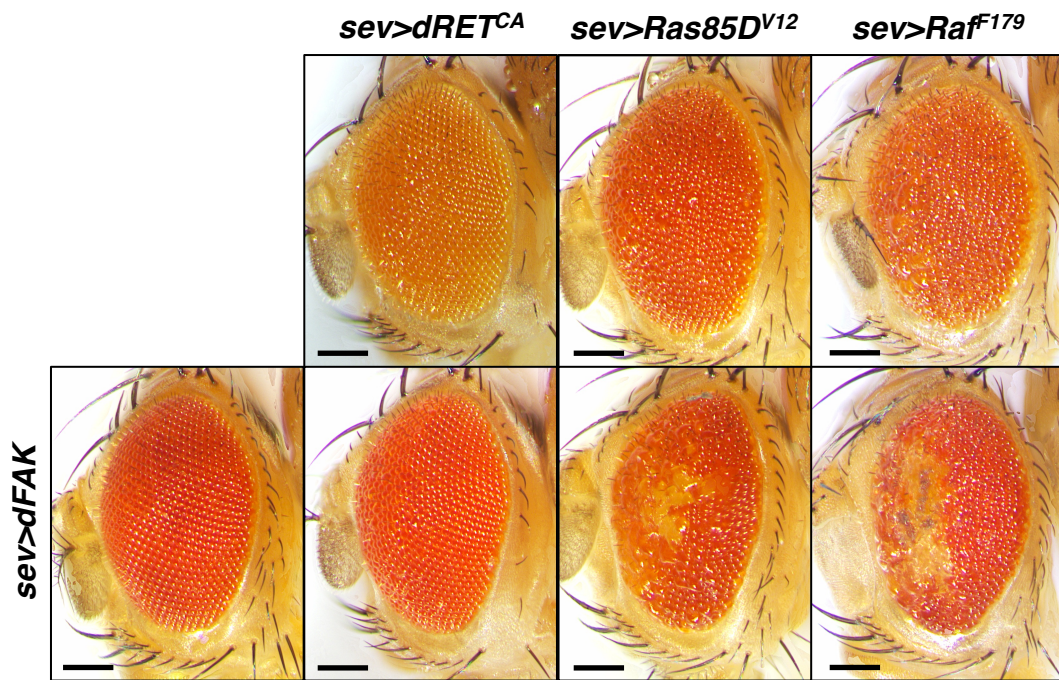

Supplement: Figure S5 — FAK does not suppress effects of RTK/MAPK intermediaries Ras and Raf. The sevenless promoter driver sev-gal4 was used to map the suppression of dFAK in the RET/MAPK pathway (GMR-gal4 resulted in pupa lethality when driving UAS-Ras85DV12 and UAS-RafF179). dRETCA-induced patterning defects were suppressed by dFAK co-expression, while the effects produced by the expression of activated isoforms of Ras (RasV12) or Raf (RafF179) were not suppressed; moreover, patches of non-pigmented ommatidia appeared when dFAK was co-expressed, implying that dFAK suppresses dRET signalling upstream of Ras in the MAPK pathway. (PDF) [file pgen.1004262.s005.pdf]

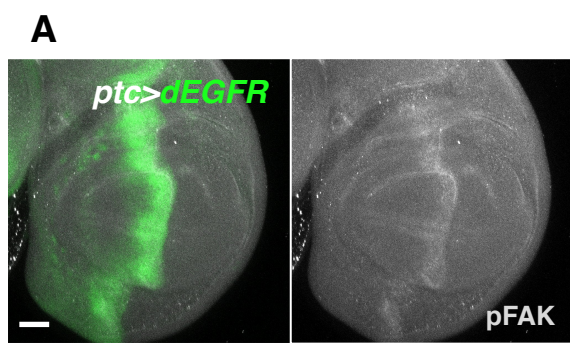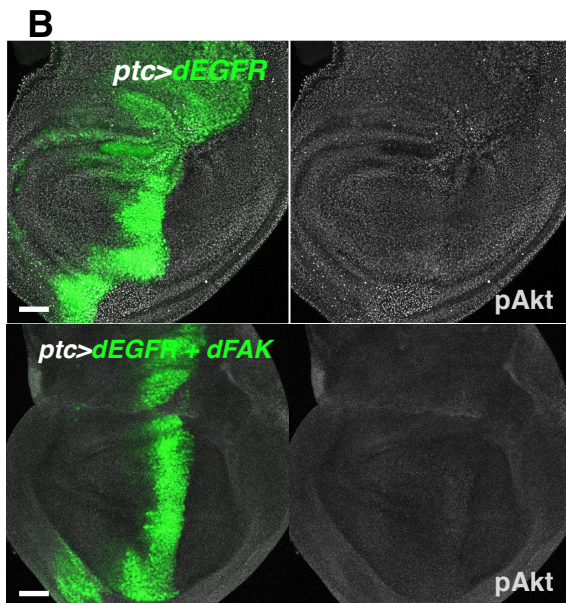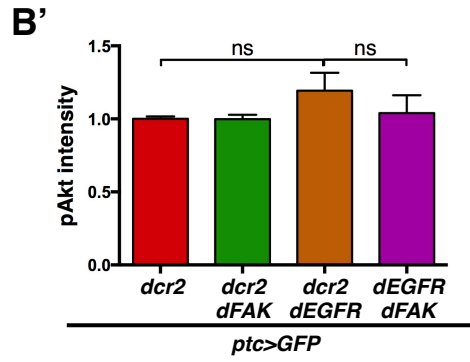

*GMR>dEGFR; GMR>*

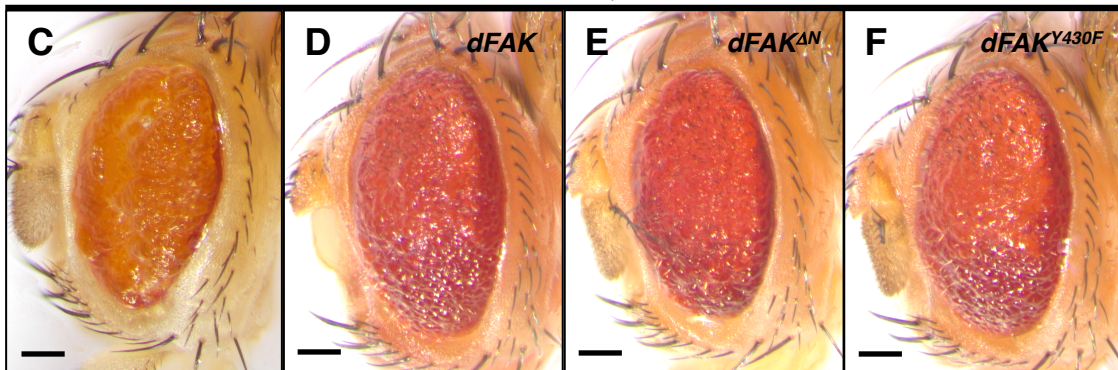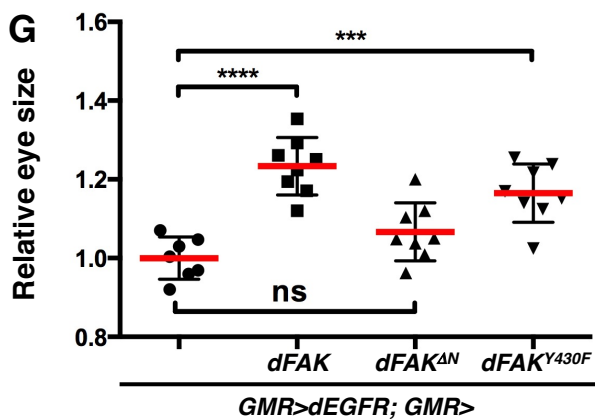

Supplement: Figure S6 — FAK suppressed the RTK dEGFR. (A) Immunostaining assays showed increase phosphorylation of dFAK upon dEGFR expression in the ptc stripe of the wing disc. (B) dEGFR did not activate Akt1 phosphorylation significantly in the ptc stripe; dFAK co-expression made no difference either, as assessed by quantification of immunostaining signal (B′). Scale bar, 50 µm. Control genotypes were shown in Figure S4A–B. (C–F) Eye micrographs show the effects of different dFAK mutant isoforms on the dEGFR-overexpression phenotype. Note that similar to the case of dRETCA (Figure 3), the N-terminus domain mutant (dFAKΔN) did not affect dEGFR reduced eye size, while a full-length dFAK and a kinase mutant dFAK isoform (dFAKY430F) did suppress this phenotype. Scale bars, 100 µm. (G) Quantification of eye sizes from the different genotypes, expressed as relative values to the mean value of control panel (C) (‘ns’: not statistically significant; *** = p<0.001; **** = p<0.0001; n = 8–10 for each genotype). (PDF) [file pgen.1004262.s006.pdf]

**A**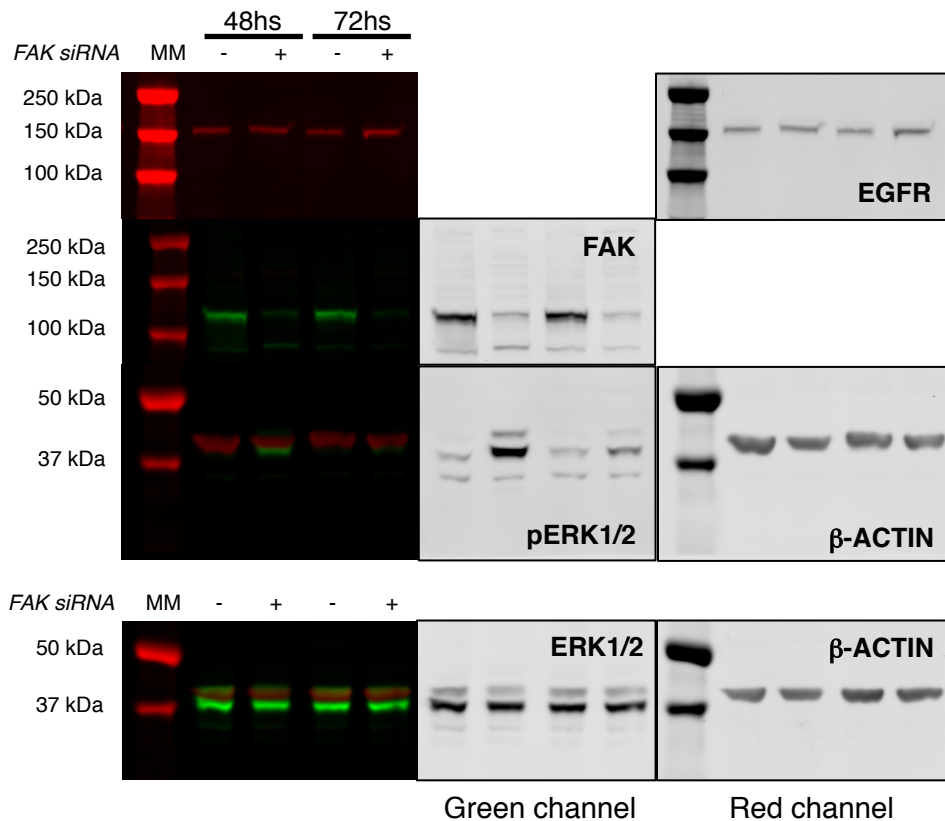**B**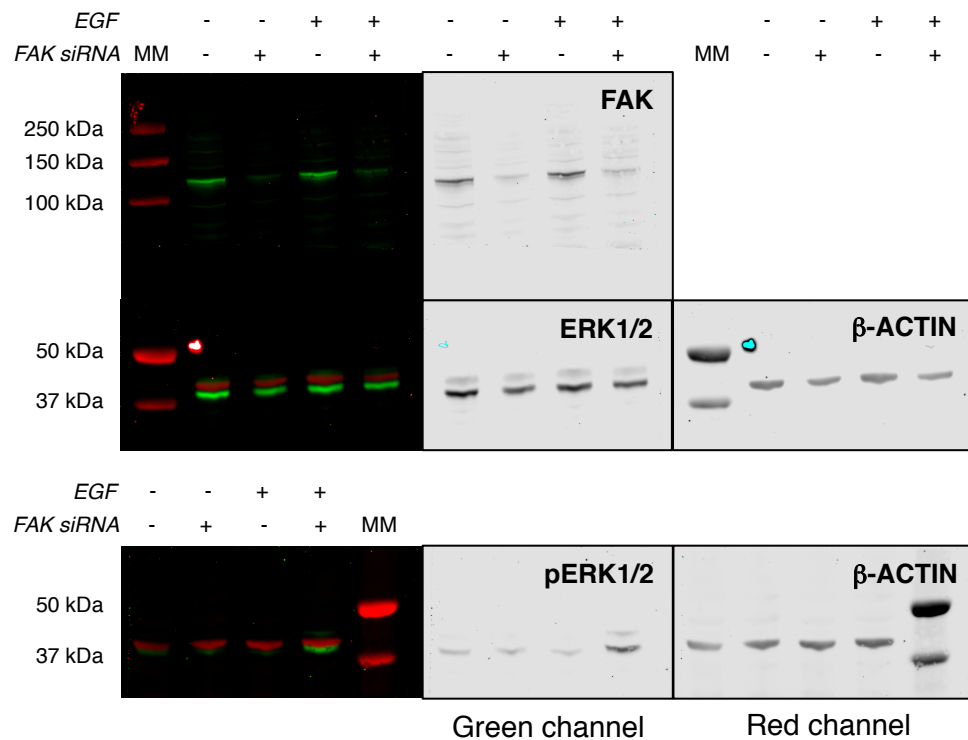

Supplement: Figure S7 — FAK negative regulation of EGFR is conserved in MDA-MB-231 human breast adenocarcinoma cells. Expansions of Figure 7G (A) and 7H (B), which shows the un-cropped western blotting images for each antibody labelling. Molecular weight markers (MM) are shown in either the first or last lanes, and the original multicolour Li-Cor scanned images are shown in the left panels. Expected molecular weights of proteins are: EGFR (175 kDa); FAK (125 kDa); ERK1/2 (44/42 kDa); β-actin (42 kDa). (PDF) [file pgen.1004262.s007.pdf]

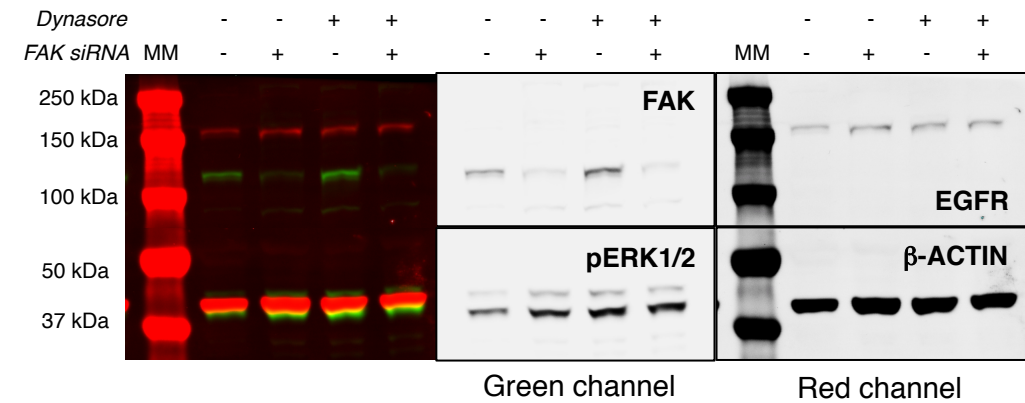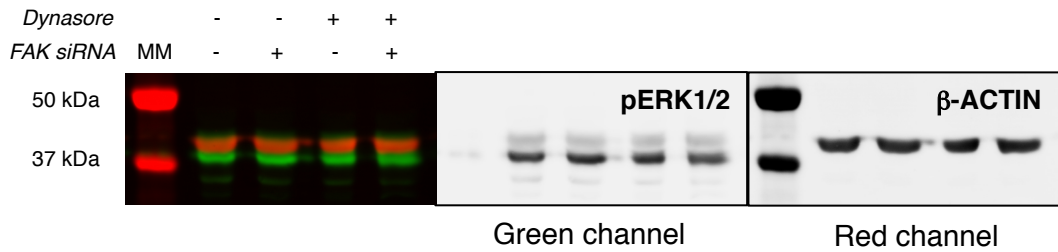

Supplement: Figure S8 — Inhibition of dynamin-dependent internalisation retains EGFR at the plasma membrane. Expansion of Figure 8D showing the un-cropped western blotting images for each antibody labelling. Molecular weight markers (MM) are shown in the first lanes, and the original multicolour Li-Cor scanned images are shown in the left panels. Expected molecular weights of proteins are: EGFR (175 kDa); FAK (125 kDa); ERK1/2 (44/42 kDa); β-actin (42 kDa). MDA-MB-231 cells were transfected with either non-targeting (siNT) or FAK-specific siRNA (siFAK) and serum starved prior to addition of 80 uM Dynasore hydrate (Sigma Aldrich). Note that siNT-transfected cells showed an increased phosphorylation of ERK1/2 in response to Dynasore treatment (80 µM, 30 minutes). (PDF) [file pgen.1004262.s008.pdf]
