# Supplementary material for: FAK Acts as a Suppressor of RTK-MAP Kinase Signalling in Drosophila melanogaster Epithelia and Human Cancer Cells
Source: PLoS Genet. 2014 Mar 27;10(3):e1004262. doi: 10.1371/journal.pgen.1004262 (PMC3967952; doi:10.1371/journal.pgen.1004262)
Supplement: Text S1 — This text file provides a list of the full genotypes used in all figures, as well as a list of primer sequences used for PCR. (DOCX) [file pgen.1004262.s009.docx]

**Macagno et al.**

**Supplementary text S1**

**Detailed genotypes of animals used in the study.**

**Figure 1**

A-C: *UAS-dicer2/+; ptc-gal4, UAS-GFP/+*

D-F: *ptc-gal4, UAS-GFP/+; UAS-dRET^CA^/+*

G: *Canton-S*

H: *GMR-dRET^WT^/+*

I: *dFAK^CG1^/+; GMR-dRET^WT^/+*

J: *dFAK^CG1^; GMR-dRET^WT^/+*

K: *GMR-gal4/+; dFAK^CG1^; GMR-dRET^WT^/UAS-dFAK*

L: *w^1118^; dFAK^CG1^*

M: *GMR-dRET^CA^/+*

N: *dFAK^CG1^/+; GMR-dRET^CA^/+*

O: *GMR-dRET^CA^*

P: *GMR-gal4/+; GMR-dRET^CA^; UAS-dFAK/+*

**Figure 2**

A: *GMR-gal4/UAS-dicer2*

B: *GMR-gal4/+; UAS-dFAK/+*

C: *GMR-dRET^WT^/+*

D: *GMR-gal4/+; GMR-dRET^WT^/UAS-dFAK*

E, I, M: *UAS-dicer2/+; ptc-gal4, UAS-GFP/+*

F, J, M: *UAS-dicer2/+; ptc-gal4, UAS-GFP/+; UAS-dFAK/+*

G, K, M: *UAS-dicer2/+; ptc-gal4, UAS-GFP/+; UAS-dRET^CA^/+*

H, L, M: *ptc-gal4, UAS-GFP/+; UAS-dFAK/UAS-dRET^CA^*

O, N: *UAS-dicer2/+; dpp-gal4, UAS-GFP/+*

P, N: *dFAK^CG1^/dFAK^5-SZ-3124^; dpp-gal4, UAS-GFP/+*

Q, N: *dpp-gal4, UAS-GFP/UAS-dRET^CA^*

R, N: *dFAK^CG1^/dFAK^5-SZ-3124^; dpp-gal4, UAS-GFP/UAS-dRET^CA^*

**Figure 3**

B: *GMR-gal4/U-dicer2*

B: *GMR-gal4/+; UAS-dFAK/+*

B: *GMR-gal4/+; UAS-dFAK^ΔN^/+*

B: *GMR-gal4/+; UAS-dFAK^Y430F^/+*

D, C: *UAS-dicer2/GMR-gal4; UAS-dRET^CA^/+*

E, C: *GMR-gal4/+; UAS-dRET^CA^/UAS-dFAK*

F, C: *GMR-gal4/+; UAS-dRET^CA^/UAS-dFAK^ΔN^*

G, C: *GMR-gal4/+; UAS-dRET^CA^/UAS-dFAK^Y430F^*

**Figure 4**

A: *w^1118^*

B: *w^1118^; dFAK^CG1^*

C: *GMR-dRET^WT^/+*

D: *w^1118^*

E: *w^1118^; dFAK^CG1^*

F: *GMR-dRET^WT^/+*

G: *GMR-gal4/+; GMR-dRET^WT^/UAS-dFAK*

H, L: *GMR-gal4/+; GMR-hid/+*

I, L: *GMR-gal4/+; GMR-hid/+; UAS-dFAK/+*

J, L: *GMR-gal4/+; GMR-hid/+; GMR-dRET^WT^/+*

K, L: *GMR-gal4/+; GMR-gal4/+; GMR-hid/+; GMR-dRET^WT^/UAS-dFAK*

**Figure 5**

A, E: Canton-S

B, F: *dFAK^CG1^; GMR-dRET^WT^/+*

C, G: *GMR-dRET^CA^*

D, H: *GMR-gal4/+; GMR-dRET^CA^; UAS-dFAK/+*

**Figure 6**

A, E: *UAS-dicer2/+; ptc-gal4, UAS-GFP/+*

B, E: *UAS-dicer2/+; ptc-gal4, UAS-GFP/+; UAS-dFAK/+*

C, E: *UAS-dicer2/+; ptc-gal4, UAS-GFP/+; UAS-dRET^CA^/+*

D, E: *ptc-gal4, UAS-GFP/+; UAS-dFAK/UAS-dRET^CA^*

**Figure 7**

A: *GMR-gal4/UAS-dEGFR; UAS-GFP/+*

B: *GMR-gal4/UAS-dEGFR; UAS-dFAK/+*

C, E, F: *UAS-dicer2/UAS-dEGFR; ptc-gal4, UAS-GFP/+*

D, E, F: *UAS-dEGFR/+; ptc-gal4, UAS-GFP/+; UAS-dFAK/+*

**Figure S1**

A:

*w^1118^; dFAK^CG1^*

*y^1^, w^67c23^; P^{y[+mDint2] w[BR.E.BR]=SUPor-P}^ dFAK^KG00304^*

*w^1118^; P^{RS5}^ dFAK^5-SZ-3124^*

C:

*Canton-S (WT)*

*GMR-dRET^WT^/+*

*dFAK^CG1^; GMR-dRET^WT^/+*

*dFAK^CG1^/dFAK^KG00304­^; GMR-dRET^WT^/+*

*dFAK^CG1^/dFAK^5-SZ-3124^; GMR-dRET^WT^/+*

D:

*Canton-S* (*WT*)

*GMR-dRET^WT^/+*

*dFAK^CG1^; GMR-dRET^WT^/+*

*GMR-gal4/+; dFAK^CG1^; GMR-dRET^WT^/UAS-dFAK*

*GMR-dRET^CA^*

*GMR-gal4/+; GMR-dRET^CA^; UAS-dFAK/+*

**Figure 2S**

A:

*GMR-gal4/+; UAS-Src42A^RNAi^/+*

*GMR-gal4/+; UAS-Ras85D^RNAi^/+*

*GMR-gal4/+; GMR-dRET^WT^/UAS-Src42A^RNAi^*

*GMR-gal4/+; GMR-dRET^WT^/UAS-Ras85D^RNAi^*

B:

*GMR-gal4/+; GMR-hid/+*

*GMR-gal4/+; GMR-hid/+; GMR-dRET^WT^/+*

*GMR-gal4/+; GMR-hid/+; GMR-dRET^WT^/UAS-Src42A^RNAi^*

*GMR-gal4/+; GMR-hid/+; GMR-dRET^WT^/UAS-Ras85D^RNAi^*

C:

*GMR-gal4/+*

*GMR-gal4/+; UAS-dFAK^Y430F^/+*

*GMR-gal4/+; dFAK^CG1^/+; GMR-dRET^WT^/+*

*GMR-gal4/+; dFAK^CG1^/+; GMR-dRET^WT^/UAS-dFAK^Y430F^*

**Figure 3S**

A-C:

*Canton-S*

*GMR-dRET^WT^/+*

*dFAK^CG1^; GMR-dRET^WT^/+*

D:

*GMR-gal4/+; UAS-Ras85D^RNAi^/+*

*GMR-gal4/+; dFAK^CG1^; GMR-dRET^WT^/UAS-Ras85D^RNAi^*

*GMR-gal4/+; GMR-dRET^CA^; UAS-Ras85D^RNAi^/+*

E:

*Canton-S*

*dFAK^CG1^; GMR-dRET^WT^/+*

**Figure 4S**

A, H, G, L: *UAS-dicer2/+; ptc-gal4, UAS-GFP/+*

B, I, G, L: *UAS-dicer2/+; ptc-gal4, UAS-GFP/+; UAS-dFAK/+*

C, J, G, L: *UAS-dicer2/+; ptc-gal4, UAS-GFP/+; UAS-dRET^CA^/+*

D, K, G, L: *ptc-gal4, UAS-GFP/+; UAS-dFAK/UAS-dRET^CA^*

E, G: *UAS-dicer2/+; ptc-gal4, UAS-GFP/UAS-dInR*

F, G: *ptc-gal4, UAS-GFP/UAS-dInR; UAS-dFAK/+*

**Figure 5S**

*UAS-dicer2/+; sev-gal4; UAS-dRET^CA^/+*

*UAS-dicer2/+; sev-gal4; UAS-Ras85D^V12^/+*

*UAS-dicer2/+; sev-gal4; UAS-dRaf^F179^/+*

*UAS-dicer2/+; sev-gal4/+; UAS-dFAK/+*

*UAS-dicer2/+; sev-gal4; UAS-dRET^CA^/UAS-dFAK*

*UAS-dicer2/+; sev-gal4; UAS-Ras85D^V12^/UAS-dFAK*

*UAS-dicer2/+; sev-gal4; UAS-dRaf^GOF^/UAS-dFAK*

**Figure 6S**

A: *UAS-dicer2/UAS-dEGFR; ptc-gal4, UAS-GFP/+*

B:

*UAS-dicer2/UAS-dEGFR; ptc-gal4, UAS-GFP/+*

*UAS-dEGFR/+; ptc-gal4, UAS-GFP/+; UAS-dFAK/+*

C, G: *GMR-gal4/UAS-dEGFR; UAS-GFP/+*

D, G: *GMR-gal4/UAS-dEGFR; UAS-dFAK/+*

E, G: *GMR-gal4/UAS-dEGFR; UAS-dFAK^ΔN^/+*

F, G: *GMR-gal4/UAS-dEGFR; UAS-dFAK^Y430F^/+*

**List of primers used for RNA quantification.**

1F: TGAAGTGAAAACACCGCAAG

1R: GCTTTCCCGACCACATTAAA

2F: CGGAAGAAGGATCACCGATA

2R: TGTCTGGGCATTCGTCATTA

3F: ACACAACGCTGGATTGTGAA

3R: TGTCGCAGAGCCAGTTAATG
